# From time-series deep learning to physics-constrained population pharmacokinetics: AI-driven prediction of drug concentrations and exposure for model-informed dosing

**DOI:** 10.3389/fphar.2026.1963219

**Published:** 2026-09-22

**Authors:** Yichao Xu, Zourong Ruan, Bo Jiang

**Affiliations:** Center of Clinical Pharmacology, The Second Affiliated Hospital of Zhejiang University, School of Medicine, Hangzhou, Zhejiang, China

**Keywords:** deep learning, model-informed precision dosing, physics-informed neural networks, population pharmacokinetics, therapeutic drug monitoring

## Abstract

Therapeutic drug monitoring (TDM) starts from a single measured concentration, which population pharmacokinetics (PopPK) with Bayesian maximum *a posteriori* (MAPB) estimation converts into a revised dose. Since roughly 2021, two research lines have been converging. Time-series deep learning models read longitudinal electronic health record (EHR) data and predict concentrations directly, led by long short-term memory (LSTM) networks, neural ordinary differential equations (neural-ODEs), and attention-based architectures. Physics-informed neural networks (PINNs), scientific machine learning (SciML), and related mechanism-aware approaches embed pharmacokinetic knowledge into neural networks, chasing accuracy without giving up interpretability. We survey both strands from 2019 to 2026, covering foundational papers, representative applications, and the open-source ecosystem. We organize the mechanism-learning relationship into three levels: loose coupling feeds pharmacokinetic-derived features into a machine learning model; medium coupling builds mechanistic structure into the architecture; tight coupling writes the mechanism into the loss function as differentiable constraints. We compare the three across predictive accuracy, extrapolation, interpretability, data needs, computational cost, and clinical acceptability. A study-level evidence map and a critical appraisal of the primary statistics underpin a nuanced picture: mechanism constraints consistently improve extrapolation and identifiability and keep the physics consistent, but in-distribution accuracy gains over well-tuned PopPK + MAPB are modest, and several headline results rest on preprints or synthetic-data validation. We also locate each study on an evidence hierarchy separating concentration prediction from dose recommendation and clinical benefit. Open problems include data sparsity, identifiability, uncertainty quantification, multi-drug generalization, regulatory acceptance, and foundation models. We close with a roadmap toward mechanism-informed, clinically deployable precision-dosing systems. Throughout, pediatric antiepileptic drug (AED) TDM, above all lacosamide, lamotrigine, and oxcarbazepine, serves as the guiding clinical scenario; narrow therapeutic windows, developmentally heterogeneous sampling, and multi-drug comorbidity make it the clearest proving ground for the coupling framework, and we map the evidence gaps for these drugs.

## 1 Introduction

Dose adjustment rests on a single measurement, the drug concentration in the blood. That is the job of therapeutic drug monitoring (TDM). The drugs that need it share one trait: narrow therapeutic windows. Think antiepileptics, tacrolimus and the other immunosuppressants, vancomycin, voriconazole. Between a subtherapeutic and a toxic concentration, the gap can be startlingly small (Khusial et al., 2023; Chen J. et al., 2025; Chen et al., 2026; Liu R. et al., 2025; Zhang et al., 2025). Give two patients the same milligram-per-kilogram dose, computed from population averages, and the outcomes diverge: one patient stays below the effective threshold, the other drifts into toxicity. TDM attacks the problem directly. It measures the concentration in the individual and adjusts the dose so that exposure stays inside the therapeutic range (Khusial et al., 2023). One level, one patient, one decision. The question is what the model should say next.

The quantitative side of TDM leans on population pharmacokinetic (PopPK) modeling with nonlinear mixed-effects (NLME) methods, followed by maximum *a posteriori* Bayesian (MAPB) estimation. A PopPK model describes the typical concentration profile of a drug in a target population: the structural model, typical parameter values, inter-individual and residual variability, and the covariates (age, body weight, renal function, drug interactions) that explain part of the variation (Lu et al., 2021a). When a single concentration comes in, MAPB fuses the population prior with that observation, and out pops an individualized exposure estimate and, from it, a dose adjustment (Sheiner et al., 1979; Sheiner and Beal, 1982). This PopPK-MAPB pipeline is the clinical standard and sits inside most commercial TDM software (Kantasiripitak et al., 2020; Fuchs et al., 2013). Its weaknesses are just as well documented. Model building is iterative and labor-intensive. Parametric assumptions about variability and covariate relationships are often violated in real clinical data. The covariate set tends to be small. And on sparse, irregularly sampled, real-world data, particularly in special populations such as children, performance can be suboptimal (Khusial et al., 2023). Pediatric dosing, in short, leaves no room for hubris. The stakes are concrete, and children are not small adults.

Out of these weaknesses grew two research streams, and both developed quickly after 2021. The first is time-series deep learning. Long short-term memory (LSTM) networks were the initial workhorse, later joined by attention-based architectures and neural ordinary differential equations (neural-ODEs). These models estimate concentration dynamics directly from longitudinal patient data with no structural model assumed, and they make full use of electronic health record (EHR) covariates (Lu et al., 2021a; Ghanbari et al., 2026; Lu et al., 2021b). Three studies set the tone. An LSTM matched NLME performance on sparsely sampled olanzapine data (Khusial et al., 2023); neural-ODE models generalized better across regimens (Lu et al., 2021a); and an EHR-scale system forecast vancomycin troughs in the intensive care unit (Ghanbari et al., 2026). The second stream runs in the opposite direction. Rather than abandoning mechanism, it constrains it. Physics-informed neural networks (PINNs) and scientific machine learning (SciML) insert compartmental structure, mass-balance ODEs, and dosing inputs into either the network architecture or the training loss, so that interpretability and mechanistic consistency survive contact with data-driven flexibility (Raissi et al., 2019; Valderrama et al., 2024; Tsiros et al., 2026; Bisht and Agarwal, 2026; Liu S. et al., 2025; Liu et al., 2026). Distributional PINNs that recover population parameter distributions from aggregate data (Tsiros et al., 2026), SciML models that learn unknown mechanisms inside classical structure (Valderrama et al., 2024), and multi-scale physiologically informed frameworks for next-generation PBPK modeling (Liu et al., 2026) mark the current edge of this stream. Foundation models, meanwhile, are absorbing ever larger corpora, and bias travels with them, so guardrails are not optional.

The two streams are drifting toward each other, but the published record has not quite caught up with that drift. Sequence models offer representational power and can absorb high-dimensional longitudinal inputs; mechanism-constrained models offer physical plausibility, extrapolation, and trust. Yet most reviews still pick one side. Machine learning reviews in pharmacometrics emphasize predictive performance (McComb et al., 2022); PINN/SciML reviews emphasize methodology (Karniadakis et al., 2021); and the two bodies of literature rarely cite each other (Section 6.2; Table 1). Several papers do show that joining mechanistic priors with deep learning pays off, whether the goal is extrapolation (Lu et al., 2021a; Valderrama et al., 2024), working with scarce data (Tsiros et al., 2026; Han et al., 2026), or diagnosing identifiability (Bisht and Agarwal, 2026). What is missing is a common frame of reference, something that situates the degree of mechanistic coupling in any given study and tells a practitioner which integration strategy fits their problem.

**TABLE 1:** Positioning of this review against existing reviews in the two research streams.

Review (year)	Scope	Streams covered	Coupling taxonomy	Evidence map + critical appraisal	Pediatric/obstetric focus
(Fuchs et al., 2013)	Benchmarking of TDM software tools	PopPK–MAPB only	No	No	No
(Kantasiripitak et al., 2020)	MIPD software tool evaluation	PopPK–MAPB only	No	Partial (software validation criteria)	No
(McComb et al., 2022)	Machine learning in pharmacometrics	ML + PopPK; temporal DL and PINN/SciML not integrated	No	Partial (general cautions)	No
(Del Valle-Moreno et al., 2023)	MIPD software scoping review	PopPK–MAPB only	No	Partial (software quality)	No
(Karniadakis et al., 2021)	Physics-informed machine learning (broad physical sciences)	PINN/SciML methodology only	No	No	No
This review	Emerging hybrid paradigms for precision dosing	Time-series DL + physics-constrained + PopPK–MAPB, unified	Yes (loose/medium/tight)	Yes (Table 2; Section 6)	Yes (pediatric AED TDM; pregnancy; Table 4)

Positioning against existing reviews. MIPD, model-informed precision dosing; DL, deep learning. Each prior review covers one side of the two-stream landscape that this review spans; none offers a mechanistic-coupling taxonomy, a stratified study-level evidence map with peer-review status and validation type, or a pediatric/obstetric application agenda. This table substantiates the novelty claim made in the Introduction and Section 6.2.

Three tasks structure the rest of the paper. Task one, covered in Sections 3 and 4 (Table 2), is a synthesis of both research streams as they stand between 2021 and 2026, covering the foundational studies, the representative applications across drugs and populations, and the methodological lessons they leave behind. Task two, in Section 5 (Figures 1, 2; Table 3), is a taxonomy of loose, medium, and tight mechanistic coupling, designed to serve a double purpose as both a map of the integration landscape and a decision aid for selecting coupling depth. Task three, in Sections 7 and 8 (Figure 3), is an inventory of the open datasets, platforms, and code that make these methods reproducible, along with the methodological and translational hurdles likely to shape the field’s next phase. Methodological concerns drive most of what follows, but the clinical context is never far away, especially pediatric AED TDM, where sparse sampling, wide inter-individual variability, and narrow therapeutic windows make the modeling choice especially consequential. The new-generation AEDs (lacosamide, lamotrigine, and oxcarbazepine) remain the most underserved. Their pharmacokinetic models are incomplete, multi-drug interactions are common, and no integrated time-series-plus-mechanism-constrained decision tool exists for them yet, a gap that motivates the framework developed below (Chen J. et al., 2025). A fourth task, in Section 6 (Figures 4, 5), converts the evidence review into a quantitative cross-study synthesis: a harmonized performance comparison, a bibliometric and cross-citation analysis of the two streams, and a critical appraisal of the primary statistics. The structure of the paper, in other words, is not arbitrary; it tracks the field, and the framework earns its keep by use.

**TABLE 2:** Comprehensive evidence map of all included primary studies and foundational methodological anchors.

Study (year)	Peer-review	Drug(s), population	Data and n	Model vs. comparator	Performance	Validation	Code	Notes
(Khusial et al., 2023)	Journal	Olanzapine; Adults (CATIE, schizophrenia)	CATIE trial data; 523 patients/1,527 samples	LSTM-ANN hybrid vs. NONMEM PopPK	RMSE 29.566 vs. 31.129 (−5.0%)	Internal (validation split)	NR	No CI/significance test around delta
(Lu et al., 2021a)	Journal	Trastuzumab emtansine (T-DM1); Adults, HER2+ breast cancer (trials)	Industry trial data; NR (trial data)	Neural-ODE vs. LSTM, LightGBM, PopPK	Best cross-regimen prediction (deltas NR)	Cross-regimen (Q3W ↔ Q1W)	NR	Deltas not quantified
(Lu et al., 2021b)	Journal	T-DM1 (concentration–platelet PD); >600 adults (trial)	Industry trial data; >600 patients	Neural-PK/PD (neural-ODE) vs. PopPK/PD model	Better temporal prediction; simulates untested regimens	Hold-out patients	NR	—
Nephrocast-V (Ghanbari et al., 2026)	Journal	Vancomycin; Adult ICU	EHR (UCSD Health); 2,205 encounters	LSTM + multi-head attention vs. Critical-care pharmacist (+Bayesian software)	MAE 3.15, RMSE 4.17 mg/L; trough attainment 55.6% vs. 56.7% (p = 0.24)	Internal test set	NR	No pre-specified non-inferiority margin; p > 0.05 ≠ equivalence
(Chen et al., 2025a)	Journal	Valproic acid; Pediatric epilepsy	Multi-center TDM; 490 (train) + external cohort	NN, MLR, RF/XGBoost/LightGBM vs. PopPK, MAPB, MLR	NN F30 > 80%; ML/NN > PopPK	External center (Beijing Children’s Hospital)	NR	—
(Chen et al., 2026)	Journal	Tacrolimus; Adult renal transplant	Single-center TDM; 83 patients/809 records	SVR (13 algorithms, SHAP) vs. Other ML algorithms	R^2^ = 0.6498; ±30% accuracy 79.03%	Internal (train/test)	NR	Single center
(Zhang et al., 2025)	Journal	Vancomycin AUC24h; Adults (Chinese hospital)	Hospital data + PopPK simulation; 246 patients; 4,000 simulated curves	XGBoost on PopPK-simulated data vs. Bayesian estimator (MAPB)	R^2^ = 0.9993 (test)	Internal validation set (simulated)	NR	CIRCULAR: test set simulated from the same PopPK model used for training
(Liu et al., 2025a)	Journal	Voriconazole; Elderly	Single-center TDM; 270 patients	Ensemble ML + PPK (CL/F) vs. Feature-ablation baselines	31 → 9 features; real-time prediction	Internal (train/test)	NR	No pharmacogenomic covariates
(Peng et al., 2014)	Journal	Oxcarbazepine (active metabolite MHD); Chinese children with epilepsy	Single-center TDM; 466 patients/840 samples	PPK (Phoenix NLME) + MAPB basis vs. —	MHD PPK with age/TBW/dose-per-weight/gender covariates	Bootstrap + predictive check	NR	Provides the PPK prior for PPK + ML fusions
(Giacometti et al., 2025)	Journal	Dalbavancin; Adults (clinical cohort)	Sparse clinical data; 218 patients	Neural-ODE (pharmacoNODE) vs. NR (see paper)	Concentration prediction on sparse data	Internal	YES (pharmacoNODE)	—
(Raissi et al., 2019)	Journal	Method foundation (ODE/PDE); n/a (benchmarks)	Benchmark problems; n/a	PINN vs. Classical PDE solvers	Establishes the PINN framework	Benchmark problems	Open lineage	Foundational method paper
(Valderrama et al., 2024)	Journal	Simulated PK (one-compartment + NN absorption); n/a (simulated)	Simulated PK; n/a	SciML (mechanism + NN) vs. Classical one-compartment PK	Accurate extrapolation (new regimens, sparsity, patients)	Extrapolation tasks (simulated)	NR	Industry; simulated data
(Tsiros et al., 2026)	Journal	Minimal PBPK, monoclonal antibody; Population (aggregated literature)	Aggregated literature data; n/a (summary statistics)	D-PINN vs. MCMC	Accuracy comparable to MCMC	Benchmark vs. MCMC on aggregated data	NR	Unlocks summary-level PK data
(Bisht and Agarwal, 2026)	Preprint (arXiv)	Chemotherapy PK (two-compartment + Michaelis-Menten); n/a (simulated)	Simulated; n/a	PINN vs. Nonlinear least squares (clinical estimator)	Matched NLS; exposed structural non-identifiability	Simulated benchmarks	Preprint code	Preprint; synthetic data
Sim2Real-PK (Han et al., 2026)	Preprint (Research Square)	Ibrutinib; daratumumab; Oncology trial participants	YODA individual-patient data; NR (YODA IPD)	Mechanism pretraining + residual correction vs. Population-level baselines	F50 32.1% → 39.9%; F20 38.8% → 51.1%	External IPD (YODA)	NR	Preprint; largest reported effects; no CI
(Liu et al., 2025b)	Preprint (arXiv)	Multi-organ PK; n/a (dataset)	Multi-organ PK dataset; NR	Dynamic GNN vs. MLP, LSTM	MAPE 15.7%; R2 = 0.9342	Internal	NR	Preprint; dataset provenance NR
(Liu et al., 2026)	Preprint (arXiv)	PBPK foundation models; n/a (synthetic)	Synthetic PBPK data; n/a	Foundation transformers + PCDM + Neural Allometry vs. Classical PBPK	Physiological violation 2.00% → 0.50%	Synthetic	NR	Preprint; synthetic data only

Comprehensive evidence map of all primary studies and foundational methodological anchors included in this review, replacing the representative-selection tables of the original submission. “Journal” = peer-reviewed journal article; “preprint” = not yet peer-reviewed as of the search date (26 August 2026). Validation type: internal = train/test or cross-validation split within one center; cross-regimen = trained on one dosing regimen and tested on another; external = tested on an independent center, dataset, or individual-patient repository. NR, not reported in the source. The critical appraisal column flags the three evidence-quality threats defined in Section 2 (circularity; missing uncertainty quantification; missing pre-specified margins) where they apply; the full appraisal is given in Section 6.3.

**FIGURE 1:**
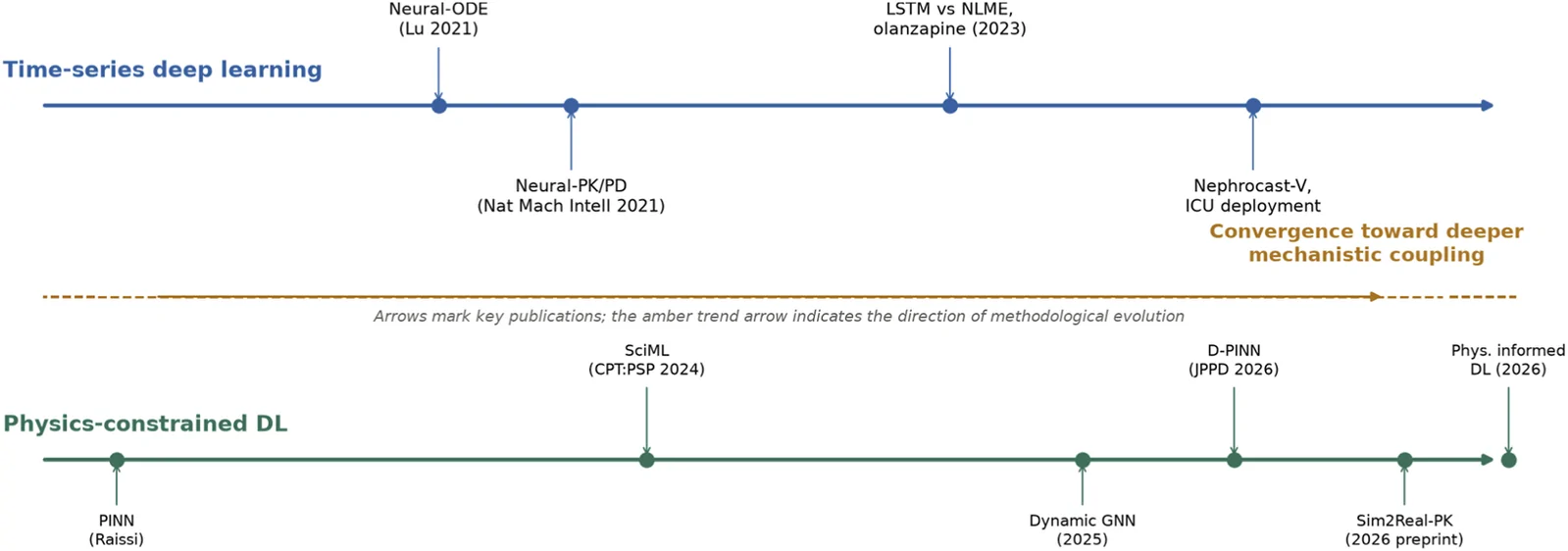
Timeline of the two research lines (2019–2026). Blue markers show time-series deep learning for drug concentration prediction; teal markers show physics-constrained deep learning for population pharmacokinetics. Circles indicate key publications with their years, and the amber dashed arrow traces the direction of methodological evolution toward progressively deeper mechanistic coupling (Section 5).

**FIGURE 2:**
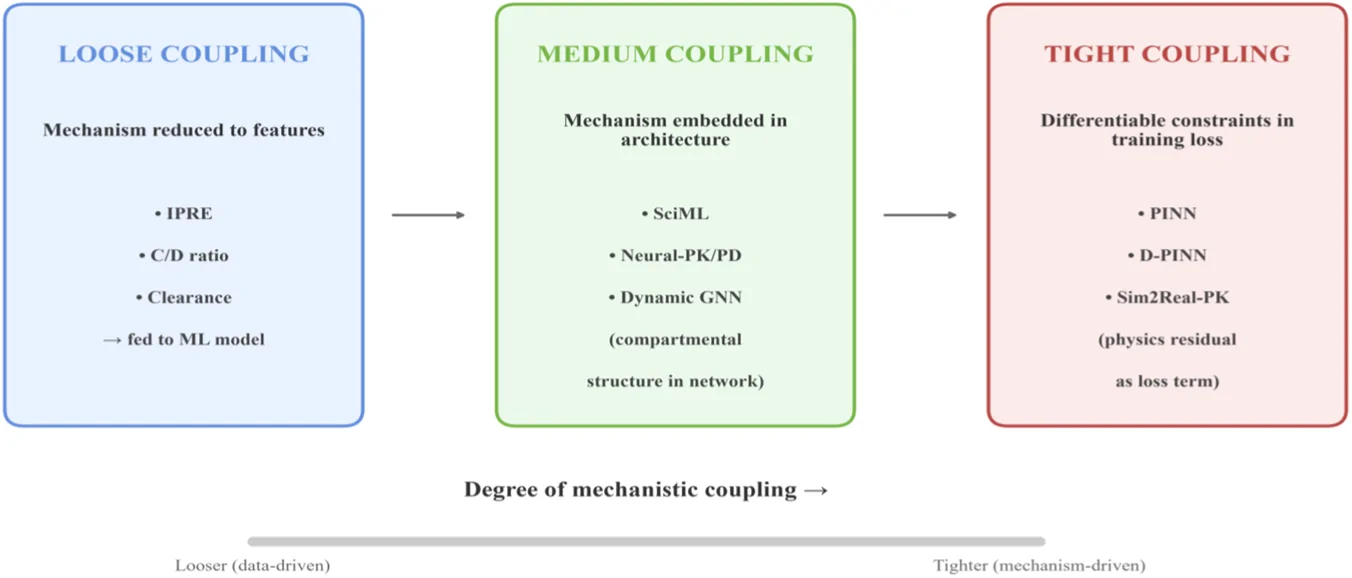
The three coupling stages. Loose: mechanism reduced to features (IPRE, C/D ratio, clearance) and fed to an ML model. Medium: mechanism embedded in the architecture (SciML, neural-PK/PD, dynamic GNN). Tight: differentiable mechanism constraints imposed on the training loss (PINN, D-PINN, Sim2Real-PK).

**TABLE 3:** How the three coupling stages compare across six dimensions.

Dimension	Loose coupling	Medium coupling	Tight coupling
Predictive accuracy (in-distribution)	Good (depends on feature quality)	Good–high	Good–high (loss balance dependent)
Extrapolation capability	Weak (training-distribution bound)	Strong (mechanism governs extrapolation)	Strong (physics anchors unseen regions)
Interpretability	Limited (features interpretable, model opaque)	High (parameters and structure keep meaning)	High (mechanism explicit; identifiability diagnosable)
Data requirements	Moderate (features aggregate info)	Moderate–high (train end-to-end)	Low–moderate (physics fills data gaps)
Computational cost	Low	Moderate–high	High (residual evaluation, loss balancing)
Clinical acceptability	High today; depends on existing infrastructure and PPK tool availability	High; mechanism interpretability helps clinicians trust outputs	Emerging; requires new evaluation and regulatory guidelines
Representative studies	(Chen et al., 2026; Liu et al., 2025a; Zhang et al., 2025)	(Lu et al., 2021b; Valderrama et al., 2024; Liu et al., 2025b)	(Raissi et al., 2019; Tsiros et al., 2026; Bisht and Agarwal, 2026; Liu et al., 2026; Han et al., 2026)

Loose, medium and tight mechanistic coupling compared on six dimensions (Section 5.4). The clinical acceptability row adds a refinement: loose coupling depends on existing hospital infrastructure and PPK, tool availability; medium coupling benefits from mechanism interpretability that supports clinician trust; tight coupling is still emerging and will need new evaluation and regulatory guidelines.

**FIGURE 3:**
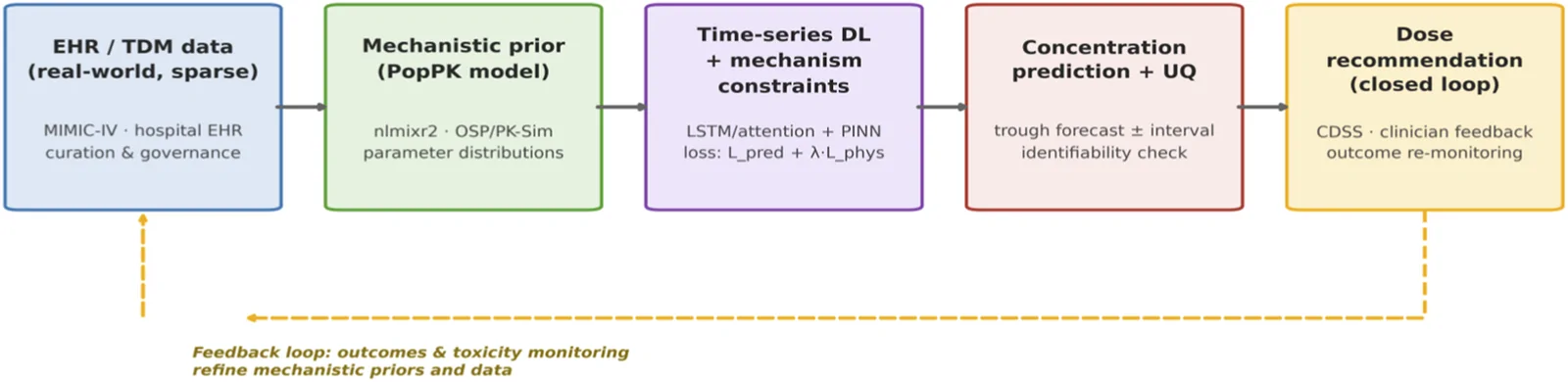
Proposed end-to-end mechanism-informed precision-dosing pipeline, illustrated for pediatric AED TDM (Sections 5.5 and 8.3). Each stage maps onto the open-source ecosystem (Section 7); validation and reporting standards are discussed in Section 8.4.

**FIGURE 4:**
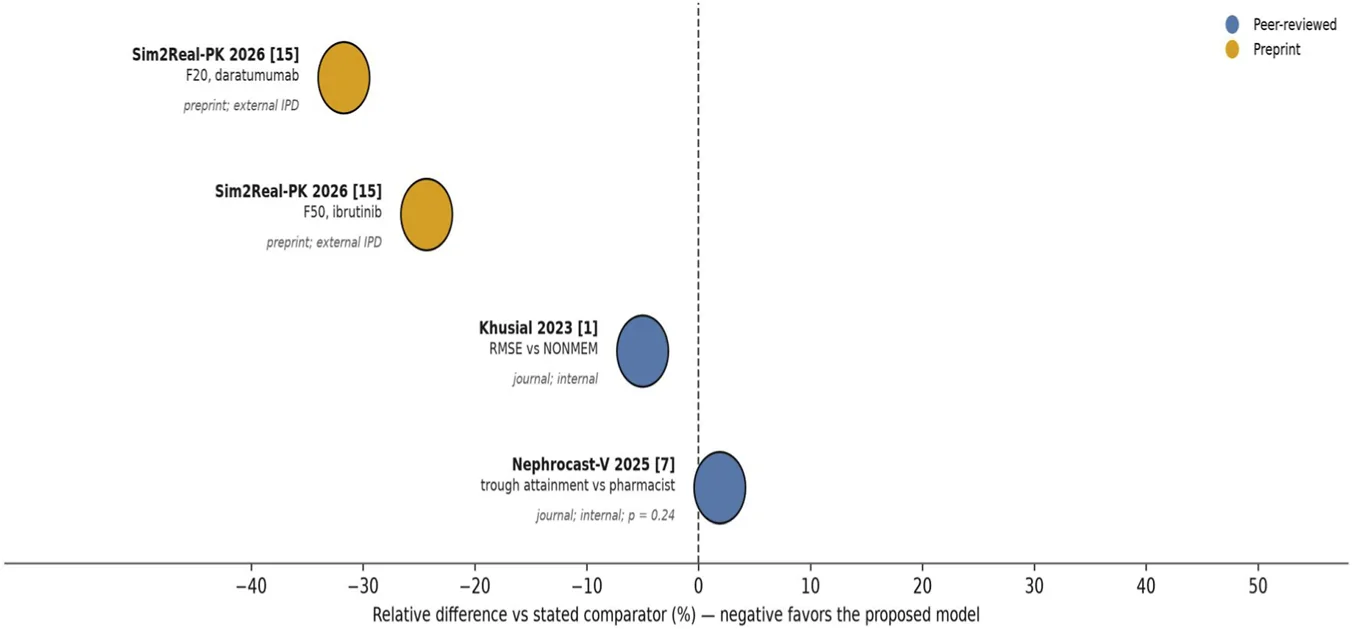
Harmonized cross-study comparison of model performance relative to the stated comparator. Effect = relative difference (%) between the proposed model and the comparator in the reported performance metric; negative values favor the proposed model. Points are annotated with peer-review status and validation type; no included study reported confidence intervals, so none are shown, and Zhang et al. (2025) is excluded because its test set was simulated from the same PopPK model used for training (circularity; Section 6.3).

**FIGURE 5:**
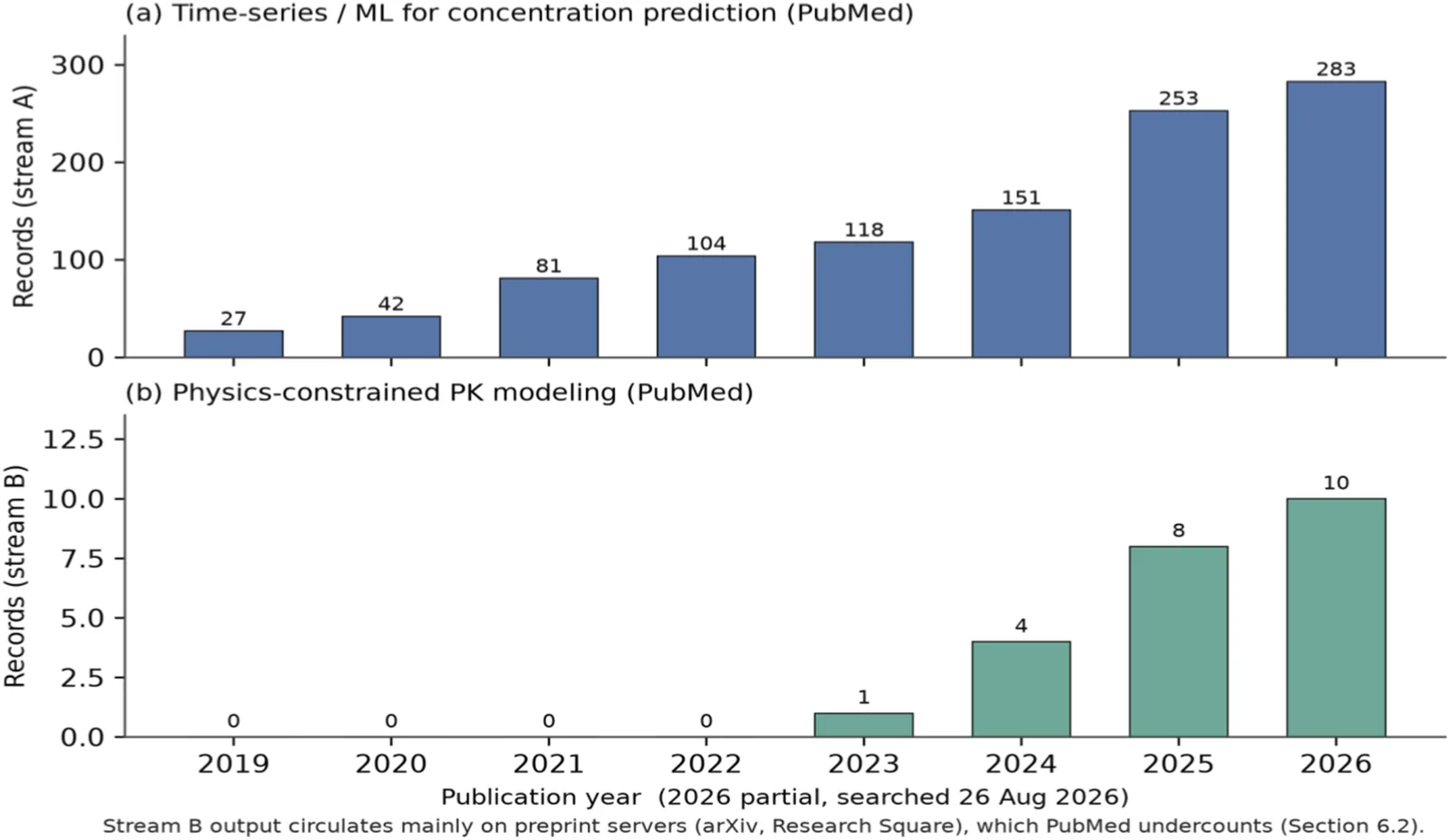
Bibliometric context: PubMed-indexed publications per year, 2019–2026 (searched 26 August 2026; 2026 partial). **(a)** The time-series/ML stream (stream A query, Section 2) grew from 27 to 283 records. **(b)** The physics-constrained stream (stream B query) remained nearly invisible in PubMed (0–10 records per year), with most activity on preprint servers; the two streams show near-zero mutual citation (Section 6.2).

## 2 Review methodology

This is a narrative review with purposive selection. To make the synthesis reproducible, we report the full search protocol here, document the search footprint (Figure 6), and provide the complete study-level evidence base in Table 2 rather than a convenience sample.

**FIGURE 6:**
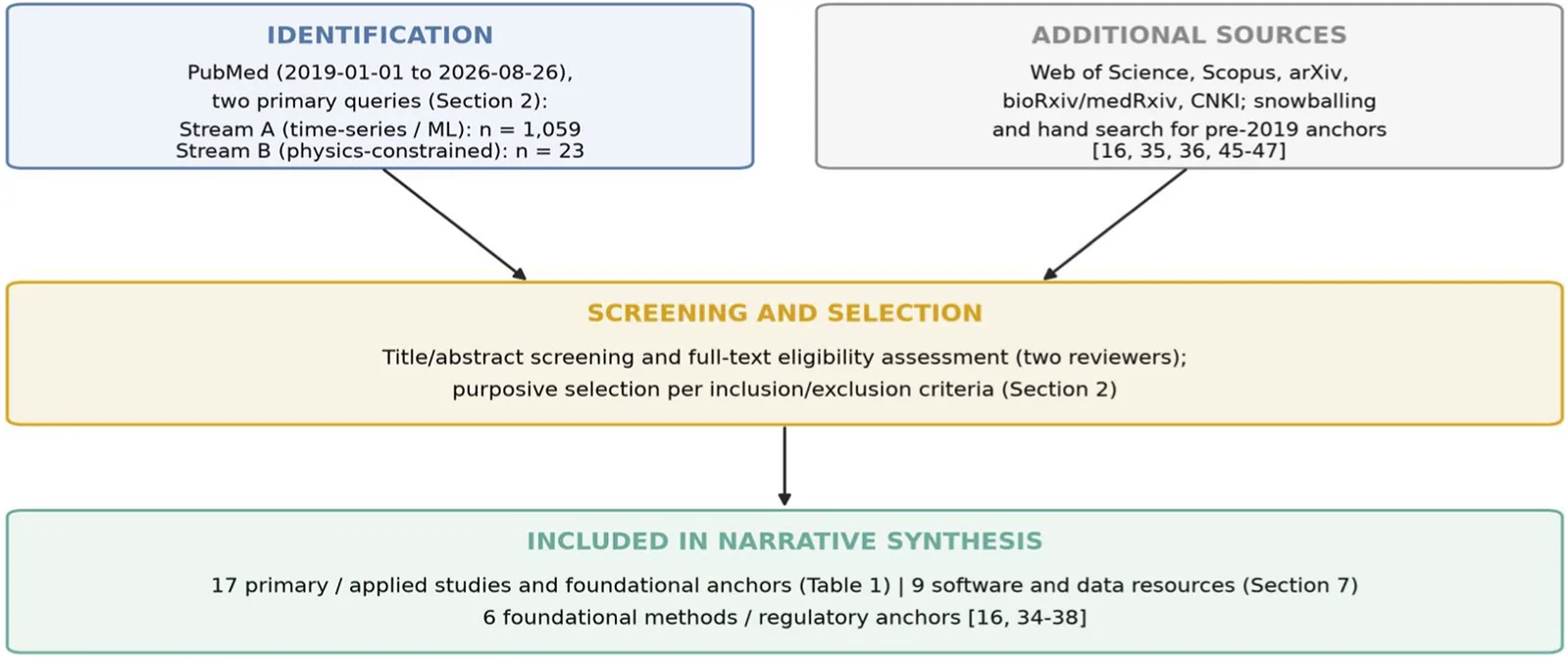
Literature search footprint (2019–2026; searched 26 August 2026). PubMed counts by search stream are exact (E-utilities); Web of Science, Scopus, arXiv, bioRxiv/medRxiv, and CNKI were searched and combined by purposive selection and snowballing (Section 2).

### 2.1 Selection principles

Four principles guided purposive selection. (i) Representativeness: the included studies cover both research streams, all three coupling levels of Section 5, and the range of drugs, populations, and data settings encountered in the search results. (ii) Methodological significance: studies that introduced an architecture, a systematic comparison, or a benchmark are prioritized over incremental applications of the same method. (iii) Evidence diversity: journal articles and preprints, international and Chinese-language work, and single-center and multi-center studies are all represented, so that peer-review status and validation breadth can be appraised rather than assumed. (iv) Extractability: only studies reporting performance metrics and validation design in extractable form populate the evidence map (Table 2). These principles favor a deliberately constructed sample over exhaustiveness, consistent with a narrative review.

### 2.2 Search strategy

We searched PubMed, Web of Science Core Collection, Scopus, arXiv, bioRxiv, medRxiv, and the China National Knowledge Infrastructure (CNKI, for Chinese-language literature) from 1 January 2019 to 26 August 2026 (the date of the final search), with no language restriction beyond English and Chinese full texts. Because the two research streams have grown in separate venues, we ran two primary PubMed queries, designed to mirror the two streams of this review:

- Stream A (time-series deep learning for drug concentration prediction): (“deep learning”[tiab] OR “machine learning”[tiab] OR “neural network”[tiab] OR “long short-term memory”[tiab] OR “neural ordinary differential equation”[tiab] OR “attention”[tiab]) AND (pharmacokinetic*[tiab] OR “drug concentration”[tiab] OR “therapeutic drug monitoring”[tiab]) AND (predict*[tiab] OR forecasting[tiab] OR dosing[tiab]).
- Stream B (physics-constrained modeling): (“physics-informed”[tiab] OR “scientific machine learning”[tiab] OR “SciML”[tiab] OR “PINN”[tiab] OR “physiologically informed”[tiab] OR “mechanism-constrained”[tiab] OR “neural-ODE”[tiab]) AND (pharmacokinetic*[tiab] OR pharmacometric*[tiab] OR “drug concentration”[tiab] OR “physiologically based”[tiab] OR “precision dosing”[tiab]).

The same queries, adapted to each database’s syntax, were run on the other databases. Reference lists of all included records were screened (snowballing) to capture foundational methodological anchors published before 2019, which the window would otherwise exclude; these include Bayesian forecasting of individual pharmacokinetics (Sheiner et al., 1979; Sheiner and Beal, 1982), neural ordinary differential equations (Chen et al., 2018), and the origin of the PINN framework (Raissi et al., 2019).

### 2.3 Eligibility criteria

We included original methodological or applied studies that (i) developed or evaluated a model for predicting drug concentrations, pharmacokinetic parameters, or exposure metrics in humans; (ii) used deep learning, machine learning, scientific machine learning, physics-informed, Gaussian process, or hybrid PopPK-ML approaches, alone or combined with population methods; (iii) concerned drugs relevant to therapeutic drug monitoring or precision dosing; and (iv) were published in peer-reviewed journals or posted as preprints, in English or Chinese, within the search window (foundational methods papers outside the window allowed via snowballing, as above). We excluded conference abstracts without full text, editorials, commentaries and letters; studies predicting pharmacokinetic parameters solely from molecular structure without concentration-time or TDM applications; purely *in vitro* or non-human applications without human relevance; and duplicate reports (for a study available both as preprint and as a peer-reviewed article, the peer-reviewed version was used; the preprint was retained only when no published version existed).

### 2.4 Screening and data extraction

Titles and abstracts were screened independently by two reviewers (Y.X., Z.R.); full texts of potentially eligible records were assessed against the inclusion criteria; disagreements were resolved by consensus with B.J. From each included primary study we extracted: study and year, peer-review status (journal vs. preprint), drug(s), population, sample size, data source, model class, comparator, performance metrics, validation type (internal split, cross-regimen, or external center/dataset), and code availability. These fields populate the complete evidence map in Table 2. Because this is a narrative review with purposive selection rather than a systematic review, a strict PRISMA flow of screening losses is not applicable; instead, Figure 6 documents the search footprint transparently, and the complete protocol (database-specific strings, search dates, and the full eligibility log) is provided in the (Supplementary Material S1).

### 2.5 Critical appraisal

In addition to summarizing performance, we appraised each included study for three pre-specified threats to evidence quality: (i) circularity, defined as training and evaluating on data generated from the same model used to create the training signal; (ii) absence of uncertainty quantification around reported performance deltas; and (iii) absence of pre-specified equivalence or non-inferiority margins in comparator studies. The results of this appraisal are reported in Table 2 (critical appraisal notes) and synthesized in Section 6.3.

## 3 Time-series deep learning for drug concentration prediction

### 3.1 Recurrent architectures and the first systematic comparisons with NLME

Recurrent networks were the first deep learning models to appear in drug concentration prediction, and the choice made sense on its face. Longitudinal data arrive as sequences, and a recurrent model processes them while a hidden state carries the relevant history forward. Long short-term memory (LSTM) networks and gated recurrent units (GRUs) dominated the early applications. Pharmacokinetic time series, however, bring their own peculiarities, including irregular sampling intervals, sparse measurements, and a long tail of clinical covariates that must be aggregated over time. The recurrent architecture proved able to absorb these well before attention-based models came along. What is worth remembering is what recurrence does and does not do. The sequence is the structure, the model reads it, but a hidden state is not the same thing as mechanism.

Khusial et al. supplied the first systematic, large-scale test, and their test case was olanzapine. They built an LSTM-ANN hybrid on 1,527 observations from 523 individuals in the CATIE study, with 11 patient-specific covariates (Khusial et al., 2023). Bayesian hyperparameter optimization yielded a validation RMSE of 29.566, just under the 31.129 of the NONMEM population model. Permutation importance singled out age, sex, and smoking status as the covariates that mattered most (Khusial et al., 2023). The performance edge itself was modest; what made the study influential was the evaluation template it established, a direct head-to-head comparison against a PopPK reference on identical data, the kind of benchmark that later work simply adopted (Khusial et al., 2023; Chen J. et al., 2025). The message landed. When a recurrent model matched and slightly beat a well-tuned NLME model on the same data, the pharmacometrics community had to take notice. Deep learning had moved from curiosity to credible alternative. The evaluation template outlived the model itself, and head-to-head comparisons became the norm.

### 3.2 Neural ordinary differential equations and continuous-time modeling

Neural ordinary differential equations (neural-ODEs) marked the second milestone. Chen et al. designed them by replacing the discrete layers of a network with the solution of an ODE parameterized by the network itself (Chen et al., 2018). Continuous-time dynamics follow naturally from that formulation, training becomes memory-efficient through the adjoint method, and, most useful of all, known dynamics can be inserted directly into the model (Chen et al., 2018). Pharmacokinetics is itself a set of differential equations, so the fit between neural-ODEs and PK problems was obvious to early adopters. The idea, in a nutshell, is that the ODE provides the prior, the network fills in the details, and continuous time meets discrete data.

Lu and colleagues took up this idea with trastuzumab emtansine (T-DM1) data from two dosing regimens, Q3W and Q1W. Their neural-ODE models predicted concentrations for an untested regimen substantially better than LSTM and LightGBM did (Lu et al., 2021a), making neural-ODEs, at that time, the most accurate method available for untested treatment regimens (Lu et al., 2021a). The deeper lesson concerns extrapolation. Continuous-time, mechanism-inspired dynamics (an ODE flow) proved decisive, while discrete-time recurrence did not, a finding that anticipates the physics-constrained line of Section 4. The paper also seeded a methodological habit that later studies kept refining, namely, training on synthetic or augmented data (here, observation-window truncation) to improve generalization (Lu et al., 2021a). New regimens probe the model, and mechanism is what buys extrapolation; data alone cannot.

The same Genentech group then extended this line to joint pharmacokinetic-pharmacodynamic modeling. Lu et al. proposed a neural-PK/PD framework that couples key pharmacological principles (dose input and state transition of the PK subsystem) with neural ODEs, and applied it to concentration-platelet response data from more than 600 patients (Lu et al., 2021b). Because the dosing mechanism was embedded in the architecture, the model improved temporal prediction relative to a state-of-the-art population PK/PD model, and it could even simulate responses to untested dosing regimens (Lu et al., 2021b). This work sits squarely at the intersection of the two lines we review. It is at once a time-series deep learning model that learns PK/PD dynamics from longitudinal data and a mechanism-embedded architecture (Section 5.2). The distinction, in other words, is a spectrum rather than a dichotomy. Data scarcity bites hardest exactly here, where every sample counts and simulation helps.

### 3.3 Attention-based models and EHR-scale clinical deployment

The move from proof-of-concept to clinical deployment is best illustrated by Nephrocast-V (Ghanbari et al., 2026). Ghanbari et al. trained a hybrid LSTM-plus-multi-head-attention architecture with skip connections on 2,205 adult ICU encounters from the University of California San Diego Health system (2016–2024), with the task of predicting vancomycin trough concentrations 2 days ahead. The results were an MAE of 3.15 mg/L and an RMSE of 4.17 mg/L (Ghanbari et al., 2026). The model consumed a rich EHR feature set (demographics, comorbidities, vital signs, laboratory measurements, medications, and prior vancomycin dosing) and tolerated missing values (Ghanbari et al., 2026). Its performance was comparable to that of a critical-care pharmacist aided by Bayesian dosing software, with therapeutic-trough attainment of 55.6% versus 56.7% and no statistically significant difference (p = 0.24). Two caveats temper this comparison. First, the original study did not pre-specify a clinical equivalence margin, and an absence of statistically significant difference is not the same as demonstrated equivalence. Second, both the model and the pharmacist attained therapeutic troughs in only slightly more than half of the cases, a rate that should itself be judged against the local standard of care before the model is adopted as a dosing tool. Notably, deviations from model-recommended doses were associated with non-therapeutic troughs (Ghanbari et al., 2026); that is the practical payoff of EHR-scale modeling. The study also offers a template for the closed-loop evaluation (prediction, recommendation, and outcome correlation) the field needs, and it highlights the engineering that clinical deployment demands, from careful cohort definition and feature engineering from raw EHR tables to an explicit reward-based evaluation of dose recommendations (Figure 7b).

**FIGURE 7:**
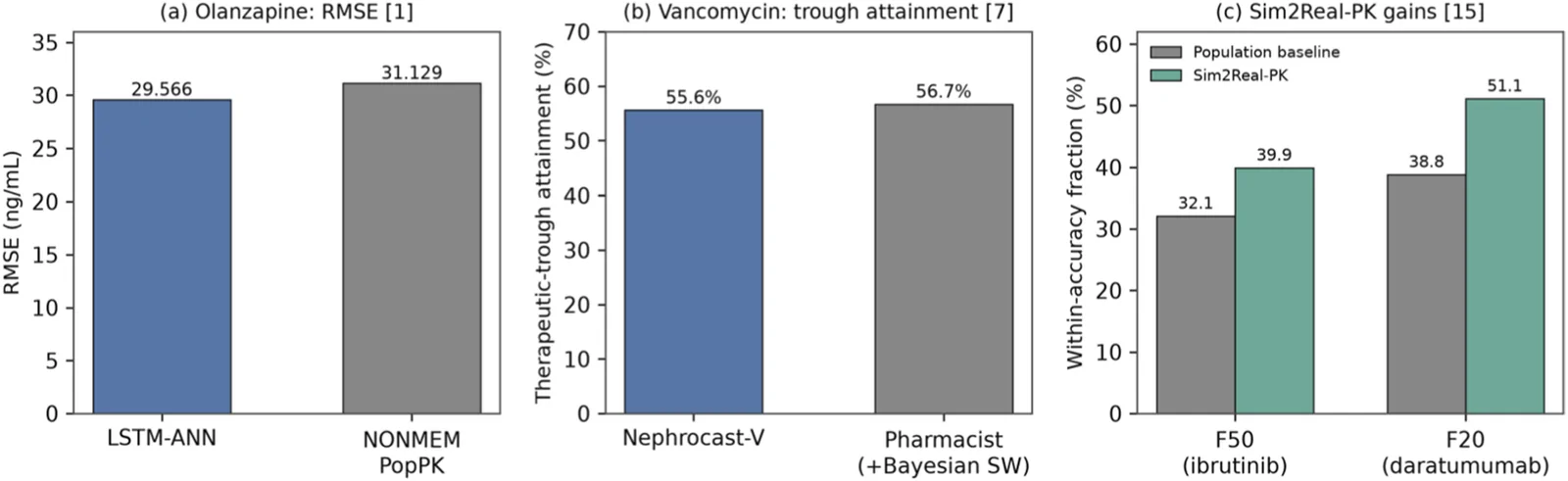
Representative performance comparisons. **(a)** Olanzapine RMSE for LSTM-ANN versus NONMEM PopPK (Khusial et al., 2023); the 5.0% difference was reported without confidence intervals (Section 6.3). **(b)** Vancomycin therapeutic-trough attainment for Nephrocast-V versus a critical-care pharmacist aided by Bayesian software (Ghanbari et al., 2026); the 1.1-percentage-point deficit (55.6% vs. 56.7%, p = 0.24) was not statistically significant, no non-inferiority margin was predefined, and both methods attained therapeutic troughs in only about 56% of cases (Section 6.3). **(c)** Precision-dosing gains of Sim2Real-PK over baseline for ibrutinib and daratumumab (Han et al., 2026) (preprint; results subject to peer review).

Beyond vancomycin, attention mechanisms and transformers are being explored for PK sequence modeling, both as standalone predictors and as components of physiologically informed frameworks (Section 4.5) (Liu et al., 2026). Self-attention captures long-range dependencies, and time-embedding strategies make it agnostic to sampling irregularity, both properties that matter for pharmacokinetic time series. Latent neural-ODE formulations, which model latent state trajectories rather than observed concentrations, have also been proposed for model-informed precision dosing (MIPD), though most remain at the preprint stage (Liu et al., 2026). The field is still early, but these architectures are the most likely next-generation of time-series models for TDM. The thread running through them is simple. Longitudinal data arrive as sequences, recurrence carries history, and attention widens the view.

A complementary, older line deserves explicit mention because it occupies the same design space as these architectures: Gaussian process (GP) and Bayesian nonparametric approaches to concentration forecasting. GPs model the concentration-time curve directly as a stochastic process, provide predictive distributions rather than point estimates, and are well suited to the small-sample, irregularly sampled regime that dominates pharmacokinetic data (Rasmussen and Williams, 2006). In the population setting, GP-based identification of typical and individual curves (treating the average response as, for example, an integrated Wiener process) was proposed well before the deep learning wave and remains a principled nonparametric alternative to NLME (Neve et al., 2007). Their current relevance to this review is threefold: GPs deliver calibrated uncertainty without ensembles or conformal post-processing; GP surrogates have been used to interpolate sparse PK curves with explicit error bars; and GP-style kernel structure can be embedded inside the mechanism-constrained models of Section 4 to quantify residual uncertainty. We flag GP methods as an under-cited bridge between classical pharmacometrics and the deep learning literature reviewed here (Section 6.2).

### 3.4 Progress in China: pediatric antiepileptic drugs and single-center PPK + ML fusion

Chinese research groups have contributed substantially to this area, especially in pediatric antiepileptic drug (AED) TDM and in combining PPK information with machine learning. Chen et al. compared PopPK, MAPB, multiple linear regression, machine learning (RF/XGBoost/LightGBM), and neural networks on valproic acid data from 490 pediatric epilepsy patients, with external validation on an independent dataset from Beijing Children’s Hospital (Chen J. et al., 2025). The original PopPK models had limited predictive performance; MAPB markedly improved predictions when prior data were available; and machine learning, especially neural networks with an F30 above 80%, reached the highest accuracy (Chen J. et al., 2025). The explicit external-validation design (training at one center, testing at another) is a methodological strength that sets this study apart from many single-center reports, and it speaks directly to the clinical motivation of this review. Pediatric AED dosing remains a domain where accurate, individualized prediction has clear and immediate benefit.

Several single-center studies illustrate a distinctive Chinese pattern of fusing PPK-derived features with machine learning (loose coupling, Section 5.1). Start with tacrolimus. A model for renal transplant recipients, built on 809 TDM records from 83 patients with 29 input variables, found SVR to be the best of 13 algorithms (R^2^ = 0.6498; ±30% accuracy 79.03%), with SHAP analysis identifying the individual-prediction exposure (IPRE) as the core predictive feature (Chen et al., 2026). Then voriconazole. A model for elderly patients combined population pharmacokinetics with an ensemble of three machine learning algorithms, cutting the input features from 31 to 9, the payoff being real-time prediction before laboratory results even become available (Liu R. et al., 2025). Then vancomycin. An AUC24h prediction model trained XGBoost on 4,000 simulated concentration-time curves generated from a PopPK model, achieving high accuracy and beating the Bayesian estimator in the validation set (Zhang et al., 2025). These studies converge on the same finding, that pharmacokinetic-derived features carry information which materially improves machine learning prediction, but the coupling remains external rather than structural. They also share a common limitation, namely, single-center, single-drug designs with limited external validation, which the international literature is beginning to address through EHR-scale studies (Ghanbari et al., 2026) and multi-center collaborations (Chen J. et al., 2025). The natural next step for this Chinese line is therefore the one pursued throughout this review, which is to keep the PPK-derived features that demonstrably help, embed the mechanism more deeply (architecturally or in the training objective), and broaden the scope from single drugs to the multi-drug pediatric AED framework analyzed in Sections 5.5 and 8.3.

A second, largely unexploited covariate source is pharmacogenomics, which the PPK + ML fusion pattern has so far ignored almost entirely. For voriconazole, *CYP2C19* genotype is a dominant determinant of exposure: poor metabolizers show approximately fourfold higher exposure than homozygous extensive metabolizers, and genotype-guided initial dosing has been shown to reduce subtherapeutic troughs and invasive fungal infections in transplant recipients (Patel et al., 2020). The elderly-patient voriconazole model reviewed above (Liu R. et al., 2025) would be a natural candidate to incorporate *CYP2C19* status as a covariate, yet none of the PPK + ML fusion studies in this review includes a pharmacogenomic input. For lamotrigine, glucuronidation by *UGT1A4* and *UGT2B7* carries a substantial genetic component (UGT1A4*3 carriers show significantly lower lamotrigine concentrations, and UGT2B7*2 and UGT2B15*2 variants further modulate exposure (Petrenaite et al., 2022)), and the interaction between UGT genotype, co-medication (valproic acid inhibition, carbamazepine induction), and developmental maturation is exactly the kind of multivariate structure that mechanistic priors could encode (Section 8.3). We regard pharmacogenomic covariates as a high-value, currently missing input channel for both the loose-coupling feature sets of Section 5.1 and the mechanistic priors of Sections 5.2, 5.3.

Taken together, the time-series line has moved from proof-of-concept parity with NLME (Khusial et al., 2023) to clinically deployable EHR-scale systems (Ghanbari et al., 2026), with neural-ODE and attention-based variants supplying continuous-time and long-range modeling capabilities (Lu et al., 2021a; Lu et al., 2021b; Liu et al., 2026). Its enduring limitation is the one we stress throughout this review. Purely data-driven sequence models excel at interpolation within the training distribution, but outside it they offer no mechanism to guarantee physically plausible behavior, precisely the regime where dosing decisions are made. That limitation is the entry point for the physics-constrained line reviewed in Section 4, and Section 5 deals with the integration of the two. Even the most capable temporal models, however, are bounded by an information ceiling set by the sampling structure itself. When a large share of patients contribute only a single trough measurement, and when within-patient variability approaches the ±30% accuracy tolerance, no model can manufacture temporal information the data never contained (Section 8.1). For pediatric AED TDM this ceiling is the rule rather than the exception, and it defines the realistic target of the coupling strategies discussed in Section 5. The lesson is not new, but it is now quantified. Pediatric dosing leaves no room for hubris, and mechanism is the missing prior.

## 4 Physics-constrained deep learning for population pharmacokinetics

### 4.1 Physics-informed neural networks: foundations

Raissi et al. introduced physics-informed neural networks (PINNs) as a way to train a network that approximates the solution of a physical system while penalizing the residual of the governing differential equations in the loss function (Raissi et al., 2019). The network receives space-time coordinates and returns the state variables; automatic differentiation produces the derivatives needed to evaluate the differential equation; and that residual (the physics loss) is added to the data-fitting loss. The trained model therefore obeys the known physics and fits the available observations at the same time. The practical upshot is that PINNs need markedly less data than purely data-driven networks, and they have a principled answer for regions with no data at all (Raissi et al., 2019). That last property is not a detail but the point. PINNs penalize equation residuals, automatic differentiation closes the loop, and the mechanism stays visible.

PINNs started in computational physics, where they were used to solve forward and inverse problems for nonlinear partial differential equations in fluid dynamics and related fields (Raissi et al., 2019). Their relevance to pharmacometrics is easy to see. Pharmacokinetics is a system of ordinary differential equations for compartmental mass balance; the mechanism is usually known in advance; and the data are sparse. A PINN enforces that known mechanism instead of learning or ignoring it. This conceptual alignment, together with the growth of the scientific machine learning (SciML) agenda (Valderrama et al., 2024), explains why physics-constrained approaches spread so quickly through pharmacometrics after roughly 2023, and it frames the applications reviewed below. The lesson, repeated across these studies, is that continuous-time flows generalize in ways discrete recurrence does not.

### 4.2 PINNs in pharmacokinetics: parameter recovery and identifiability

A landmark application is the distributional PINN (D-PINN) of Tsiros et al. (Tsiros et al., 2026). A D-PINN uses a dual-output network to predict the mean and variance of concentration over time, and couples those predictions to the pharmacokinetic ODE system through a sampling-based residual loss. In doing so, it recovers population-level parameter distributions from published summary statistics alone, namely, means and variances (Tsiros et al., 2026). The methodology accounts explicitly for inter-individual variability through the parameter distribution and for measurement noise through a residual error model, and it operates on aggregated literature data, an important capability for data-scarce settings where individual-level records are unavailable (Tsiros et al., 2026). Validated on a minimal PBPK model for monoclonal antibodies and benchmarked against Markov chain Monte Carlo, the D-PINN achieved comparable accuracy using only summary statistics (Tsiros et al., 2026). That is a significant methodological contribution, because it unlocks decades of published mean-concentration profiles that classical NLME analysis cannot exploit without individual data. Benchmarks against MCMC at this level remain rare and valuable.

Bisht and Agarwal examined PINNs in chemotherapy pharmacokinetics (preprint; citation to be updated if formally published) (Bisht and Agarwal, 2026). On a linear two-compartment model, the PINN matched the clinical nonlinear least-squares estimator and, in the same training pass, recovered the unobserved tissue concentration curve, something classical estimators cannot do because the tissue compartment is never measured. Moving to a Michaelis-Menten extension exposed the value of the approach even more clearly. The PINN openly revealed a structural non-identifiability that plasma-only data cannot resolve, while the biexponential closed-form estimator silently returned meaningless rate constants; adding just two sparse tissue observations largely restored identifiability (Bisht and Agarwal, 2026). The general point is that PINNs are more than estimators. They double as diagnostic instruments, surfacing identifiability problems that classical methods quietly ignore, a property with direct implications for trustworthy modeling (Section 8.2).

### 4.3 Scientific machine learning and mechanism-embedded architectures

A different line within SciML keeps the mechanistic structure intact and learns only the parts that are unknown. Valderrama et al. (AbbVie and Fraunhofer SCAI) made the case in an influential industry study. The design was a one-compartment PK model in which only the absorption process is learned by a neural network, while distribution and elimination parameters are estimated classically. Extrapolation tasks covered new dosing regimens, different sampling sparsity, and entirely new patients, and concentrations were predicted accurately (Valderrama et al., 2024). Because the compartmental structure was preserved and only the unknown mechanism was learned, the model kept both the accuracy and the interpretability of classical models (Valderrama et al., 2024). This structure-plus-neural-component pattern is the prototype of medium coupling (Section 5.2), and it carries a design principle that has since become widespread, to retain what is mechanistically known, learn what is not, and judge the model on extrapolation rather than interpolation. The recipe is simple (structure first, learning second), and extrapolation is what judges the result.

Deciding which component to learn is itself a modeling choice that trades flexibility against identifiability. Learning an absorption process with a flexible network captures complex, non-parametric absorption profiles that classical models can only represent with restrictive functional forms. At the same time, the learned component inherits the data-hunger and potential overfitting of neural networks, which the mechanistic anchors help to control (Valderrama et al., 2024). This is precisely how SciML bridges the two lines of this review, drawing on the representational capacity of deep learning but disciplined by mechanism. That discipline is the whole point. The network learns what the mechanism leaves open, and flexibility is bought, not free.

### 4.4 Mechanism pretraining with residual correction

A newer approach, exemplified by Sim2Real-PK (a preprint on Research Square as of the date of this review; the citation should be updated once peer-reviewed) (Han et al., 2026), separates the learning of universal kinetic laws from adaptation to clinical heterogeneity (Han et al., 2026). The framework recasts pharmacokinetic forecasting as a time-aware latent state-space trajectory inference problem. It first pretrains a neural dynamics core on large synthetic datasets generated from mechanistic ODE systems, so that the network internalizes universal kinetic laws (the Sim phase); it then adapts to real clinical data through a constrained residual-correction pathway that accounts for patient-specific deviations (the Real phase) (Han et al., 2026). Two phases, one goal. Simulation teaches, reality corrects, and the gap closes. Sim2Real-PK is not itself a foundation model. It adopts the pretrain-then-adapt recipe characteristic of foundation-model training, but its pretraining corpus consists of synthetic mechanistic simulations rather than broad clinical corpora.

Sim2Real-PK was tested on individual participant data from the YODA Project for two drugs with different pharmacological profiles, ibrutinib and daratumumab, and clearly beat conventional population-level baselines. F50 for ibrutinib rose from 32.1% to 39.9%; F20 for daratumumab, from 38.8% to 51.1%. The model recovered the expected linear and nonlinear pharmacological behaviors and resolved latent pharmacokinetic heterogeneity that traditional analytical frameworks had left obscured (Han et al., 2026) (Figure 7c). The recipe of synthetic pretraining plus residual correction is a strong answer to sparse clinical data and a concrete realization of tight coupling (Section 5.3). One caveat applies. The work is a preprint, not yet peer-reviewed, so its quantitative results should be read with that in mind until a formal version appears (Han et al., 2026).

### 4.5 Toward physiologically informed, graph-based and generative models

The frontier of physics-constrained PK modeling is expanding toward whole-body physiological structure, where the mechanism is not a simple compartmental ODE but the interconnected physiology of the organism. Dynamic graph neural networks (GNNs) model inter-organ interactions through recurrent message passing on a physiological graph, explicitly representing organs as nodes and their interactions as edges. On multi-organ pharmacokinetic data they achieved the lowest MAPE (15.7%) and R^2^ = 0.9342 across MLP and LSTM baselines (Liu S. et al., 2025) (a preprint at the time of writing). The graph structure itself is the mechanistic prior. Rather than learning organ interactions from scratch, the model is told which organs communicate and learns how they communicate. The study underscores the importance of structure-aware modeling for PBPK applications and offers an equation-free, data-driven route that scales beyond the assumptions of classical PBPK (Liu S. et al., 2025). Organs are nodes, edges are flows, and structure is the prior.

Liu, Qiu, and Wang proposed a multi-scale physiologically informed deep learning framework with three components. Foundation PBPK Transformers treat pharmacokinetic forecasting as sequence modeling, bringing foundation-model scale to PK. Physiologically Constrained Diffusion Models (PCDM) generate biologically compliant virtual patient populations with a physics-informed loss, reducing physiological violation rates from 2.00% to 0.50% on synthetic data. Neural Allometry, a GNN-plus-neural-ODE hybrid, learns continuous cross-species scaling laws (Liu et al., 2026). Although evaluated on synthetic data, the framework points to a future in which mechanistic constraints, generative modeling, and foundation-model scale are unified, a direction we discuss further in Section 8.5. Collectively, the studies in this subsection show that the physics-constrained line is no longer limited to compartmental ODEs; it now encompasses whole-body physiology, generative modeling, and foundation-scale architectures.

Across this line, a consistent methodological message emerges. The value of mechanism is greatest precisely where data are weakest. PINN-family methods recover parameters from aggregated or sparse data (Tsiros et al., 2026), expose identifiability (Bisht and Agarwal, 2026), preserve interpretability in extrapolation (Valderrama et al., 2024), and impose physiological consistency in generative settings (Liu et al., 2026). The synthetic-pretraining approach, meanwhile, turns mechanism into a data source in its own right (Han et al., 2026). The costs, including loss balancing, computational burden, and sensitivity to mechanistic misspecification, are real but addressable. The message is consistent, and the field is listening. Mechanism matters most when data are thin. The decisive open question, which we take up in Section 8, is not whether these methods can outperform classical estimators on benchmark tasks, but how to make them reliable and accepted enough for routine clinical and regulatory use.

## 5 A conceptual framework: from loose to tight mechanistic coupling

The studies reviewed so far suggest a simple way to organize them, which is to classify each integration of pharmacokinetic mechanism and deep learning by where the mechanistic knowledge enters the pipeline. We distinguish three stages accordingly (Figure 2). Loose coupling means the mechanism is present only as precomputed features; medium coupling means it shapes the architecture; tight coupling means it appears as differentiable constraints in the training objective. The taxonomy is descriptive, not prescriptive. Its purpose is to give the field a shared vocabulary, to make performance differences interpretable, and to help practitioners choose. In what follows, every study from Sections 3 and 4 is mapped onto one of the stages, and for each stage we discuss the mechanism involved, representative implementations, strengths, limitations, and the conditions under which it works best. The trade-offs are real. Loose coupling is cheap and tight coupling is principled, but choosing between them is rarely a free lunch.

### 5.1 Loose coupling: pharmacokinetic-derived features as machine learning inputs

Loose coupling starts by fitting a PopPK or PPK model, then extracts individual-level quantities from it, such as the individual predicted exposure (IPRE), the concentration-to-dose (C/D) ratio, or the model-predicted individual clearance, and feeds those into a separate machine learning model as input features. Mechanism reaches the learner only through these scalar summaries; the learner itself is an ordinary regression or ensemble model that never sees the dose-time history or the mechanistic equations. The appeal of this design is practical. No architectural changes are needed, validated PPK infrastructure is reused, and any off-the-shelf algorithm can be plugged in. The question that shapes everything downstream is a simple one, namely, where the mechanism enters, through features, architecture, or the loss.

Examples of the pattern are easy to find. In Chen et al.'s tacrolimus study, the SVR model that won among 13 algorithms (R^2^ = 0.6498, ±30% accuracy 79.03%) leaned on IPRE, a PPK-derived feature, as its most important predictor according to SHAP analysis, ahead of the raw clinical covariates (Chen et al., 2026). Zhang et al.'s vancomycin AUC24h model went further and treated the PopPK model as a data generator, simulating 4,000 concentration-time curves from the fitted population model, computing reference AUC24h values by the trapezoidal rule, and training an XGBoost algorithm on that synthetic but mechanistically informed dataset. The result beat the Bayesian estimator in external validation (Zhang et al., 2025). In Liu et al.'s voriconazole study, the PPK-estimated clearance (CL/F) ranked first in SHAP importance in the final ensemble, and feature selection cut the inputs from 31 to 9 while keeping the most informative PPK-derived and clinical variables (Liu R. et al., 2025). The oxcarbazepine MHD line of the Guangzhou group exemplifies the same pattern one level removed: Peng et al. established a PPK model of the active metabolite MHD in 466 Chinese children with epilepsy, and the PPK + MAPB-estimated individual clearance from this model has served as the training target for subsequent PPK + ML fusions (Peng et al., 2014). Across all of these, a quieter theme runs. Public benchmarks raise the bar, versioned code survives, and openness pays twice.

Loose coupling has real operational advantages. It is simple, modular, computationally cheap, and deployable wherever a PopPK tool and a standard ML library already exist, which is why most Chinese single-center TDM studies in Section 3.4 adopted it (Chen et al., 2026; Liu R. et al., 2025; Zhang et al., 2025). Its drawbacks are just as clear. The coupling is external and lossy. A rich mechanistic prior covering full dose-response dynamics, nonlinearities, and covariate structure is compressed into a few scalars, and information a joint model could exploit is thrown away. Errors in the PPK step (model misspecification or biased parameter estimates) pass silently into the learner, and the two stages have no way to correct each other. Extrapolation to untested regimens or populations is generally weak, because the features were learned from the training distribution alone. In short, loose coupling suits well-characterized drugs, stable clinical settings, and tasks where a small number of mechanistic summaries dominate prediction, such as trough-level forecasting in chronic dosing (Chen et al., 2026). The trade-off is plain. It is cheap to run and hard to extend.

### 5.2 Medium coupling: mechanistic structure embedded in the architecture

Medium coupling moves the mechanism from the feature level into the architecture itself. Compartmental state variables, ODE flows, dose-input ports, and physiological connectivity become part of the model, and neural components are left to learn only what the mechanism does not specify. Because the architecture can only generate biologically plausible dynamics, structural semantics survive. Parameters keep their meaning, mechanisms stay inspectable, and extrapolation follows the mechanism rather than the training distribution. The architecture is the constraint; learning fills the gaps. The real exam is dosing a patient you never saw, and structure decides the grade.

Three representative designs span the range of medium coupling. The SciML model of Valderrama et al. (AbbVie/Fraunhofer SCAI) keeps a one-compartment PK model and replaces only the unknown absorption process with a neural network, while distribution and elimination parameters are estimated classically; in extrapolation tasks covering new dosing regimens, different sampling sparsity, and entirely new patients, it retained high accuracy and the interpretability of classical compartmental models (Valderrama et al., 2024). The neural-PK/PD framework of Lu et al. embeds dose input and PK state transitions into a neural-ODE network and learns the PK/PD vector fields from data; because the dosing mechanism is structurally encoded, the model can simulate patient responses to untested dosing regimens, something purely data-driven sequence models cannot do (Lu et al., 2021b). At the level of whole-body physiology, dynamic GNNs impose the organ interaction structure as a graph and perform recurrent message passing over it, achieving the lowest MAPE (15.7%) and R^2^ = 0.9342 among MLP, LSTM, and GNN baselines on multi-organ pharmacokinetic data; the graph connectivity itself is the mechanistic prior (Liu S. et al., 2025). Three designs, three flavors of the same idea. The mechanism stays in the skeleton, and the skeleton stays mechanistic.

Across studies, medium coupling delivers the most favorable accuracy-interpretability trade-off for extrapolation tasks, and it is the natural choice when the mechanism is only partially known, which is typically the case in pharmacometrics. Compartmental structure and physiological connectivity are well established, but specific processes such as absorption, transport, or drug-specific kinetics remain uncertain (Lu et al., 2021b; Valderrama et al., 2024; Liu S. et al., 2025). Its costs are design effort and expertise. Constructing a mechanism-embedded architecture requires pharmacometricians and deep-learning engineers to collaborate closely, and the fixed mechanistic components can become a bottleneck if they are misspecified. Medium-coupled models are also usually trained for a specific structural hypothesis, which complicates comparisons across studies with different architectures. As a summary criterion, in medium coupling the mechanism acts as a fixed, non-differentiable structural constraint that defines the skeleton of the model, shaping what the model can and cannot represent, whereas the optimization objective remains data fitting. The mechanism is not part of the loss and does not compete with the data term during training (Lu et al., 2021b; Valderrama et al., 2024; Liu S. et al., 2025).

### 5.3 Tight coupling: differentiable mechanistic constraints in the training objective

In tight coupling, the mechanism enters the loss function as differentiable constraints. The network is trained simultaneously to fit the observed data and to minimize the residual of the governing differential equations (and, where relevant, boundary or positivity conditions) over a set of collocation points. This is the defining idea of physics-informed neural networks (PINNs) (Raissi et al., 2019), and its pharmacokinetic realizations include D-PINNs (Tsiros et al., 2026), the chemotherapy PK PINN of Bisht and Agarwal (Bisht and Agarwal, 2026), the constrained residual-correction pathway of Sim2Real-PK (Han et al., 2026), and the physics-informed loss of the physiologically constrained diffusion models (PCDM) in (Liu et al., 2026). The list is short but growing, and it comes with a reminder. Scale without mechanism is risky, pretraining is a hypothesis, and fine-tuning is not free.

Tight coupling offers three advantages the other stages cannot match. First, physical consistency is guaranteed in the loss, so predictions in data-sparse regions are anchored by mechanism rather than extrapolated from patterns, a decisive property for narrow-therapeutic-window drugs and for pediatric populations where sampling is intrinsically limited (Tsiros et al., 2026; Bisht and Agarwal, 2026). Second, tight coupling uniquely supports identifiability diagnostics, because the network must satisfy the mechanism, and failures or flat regions of the loss surface reveal what the data cannot determine. Bisht and Agarwal showed this explicitly. On a Michaelis-Menten two-compartment model, the PINN honestly exposed that plasma-only data leave the system structurally non-identifiable (k12 → 0), and adding two sparse tissue observations largely restored identifiability, a recovery the classical biexponential estimator could not even attempt (Bisht and Agarwal, 2026). Third, the constraint framework is compositional. Distributional assumptions (D-PINN) (Tsiros et al., 2026), latent dynamics (Sim2Real-PK) (Han et al., 2026), and generative modeling (PCDM, which reduced physiological violation rates from 2.00% to 0.50%) (Liu et al., 2026) can all be layered onto the same physics-loss backbone. Three advantages, each earned by constraint.

The costs are equally real. Balancing data-fit and physics residuals requires careful loss weighting, and poorly weighted constraints can degrade either accuracy or physical consistency. Training is computationally more expensive than a standard regression, and the approach is only as correct as the mechanistic model it encodes. A wrong mechanism enforced as a hard constraint can do more harm than a flexible learner. Tight coupling is therefore most appropriate for data-scarce, safety-critical applications, for regulatory-facing mechanism-consistent AI, and for scientific discovery tasks where the goal includes testing mechanistic hypotheses rather than only predicting concentrations (Raissi et al., 2019; Tsiros et al., 2026; Bisht and Agarwal, 2026). As a summary criterion, in tight coupling the mechanism enters the optimization as differentiable, soft constraints in the loss function. The model must satisfy both the data term and the physics residual, and the two objectives are balanced against each other during training, so the mechanism is a competitor to, rather than a container for, data fitting (Raissi et al., 2019; Tsiros et al., 2026; Bisht and Agarwal, 2026). The trade-off is real. Constraints cost compute, but they also buy trust.

### 5.4 Comparative synthesis

Table 3 sets the three stages side by side along six dimensions, namely, predictive accuracy on in-distribution data, extrapolation capability to untested regimens or populations, interpretability of predictions and parameters, data requirements for stable training, computational cost, and clinical acceptability. No single stage wins everywhere. Loose coupling is the most deployable and already clinically valuable, but it has the weakest extrapolation and interpretability of the three (Chen et al., 2026; Liu R. et al., 2025; Zhang et al., 2025). Medium coupling gives the best accuracy-interpretability balance for extrapolation tasks and is the natural default when the mechanism is only partially known (Lu et al., 2021b; Valderrama et al., 2024; Liu S. et al., 2025). Tight coupling is the most attractive for data-scarce, safety-critical, and regulatory-facing settings, provided its engineering challenges (loss balancing, cost, and mechanism correctness) are solved (Raissi et al., 2019; Tsiros et al., 2026; Bisht and Agarwal, 2026; Liu et al., 2026; Han et al., 2026).

The three stages are not mutually exclusive, and several landmark studies are best understood as hybrids. Sim2Real-PK combines synthetic pretraining on mechanistic ODE systems (a medium-coupling element) with a constrained residual-correction pathway (a tight-coupling element) (Han et al., 2026). D-PINN couples distributional assumptions directly into the optimization while the physics residual is computed on sampled trajectories (Tsiros et al., 2026). Neural-PK/PD embeds mechanism structurally (medium) and is trained end-to-end on real clinical data (Lu et al., 2021b). We therefore expect hybrid designs to dominate the next-generation of precision-dosing systems, with mechanistic structure where knowledge is confident, constraint-based losses where physical laws must hold, and learned components where uncertainty dominates. A practical corollary follows. To make these choices evidence-based rather than fashion-driven, the field needs head-to-head comparisons on identical real-world cohorts, placing PopPK + MAPB, cross-sectional ML, temporal deep learning, and a mechanism-constrained hybrid on the same data and reporting the same metrics. The comparisons that exist are preliminary but instructive. Khusial et al. pit their LSTM-ANN directly against NONMEM on identical olanzapine data (Khusial et al., 2023); Lu et al. evaluate neural-ODEs against LSTM and LightGBM across untested dosing regimens (Lu et al., 2021a); and Nephrocast-V compares model recommendations with pharmacist practice on real ICU data (Ghanbari et al., 2026). Each is a head-to-head benchmark in miniature. What is still rare, and exactly what will let the coupling taxonomy be tested rather than asserted, are systematic benchmarks that place all three coupling depths on identical cohorts. Those are what will tell a clinical pharmacology group which integration depth to adopt for a given drug and population.

### 5.5 A decision guide: choosing the coupling depth

Which stage a project should adopt depends on four factors, the data scale and sparsity, the completeness and confidence of the mechanistic knowledge for the drug and population in question, the task type (in-distribution prediction, extrapolation, or mechanism discovery), and the regulatory and clinical context (Figure 8). The rule of thumb is short. For large, dense datasets and well-characterized drugs, loose or medium coupling suffices and implementation cost stays low (Chen et al., 2026; Liu R. et al., 2025; Zhang et al., 2025). For sparse data with strong mechanistic priors, tight coupling exploits the mechanism to fill the gaps (Tsiros et al., 2026; Bisht and Agarwal, 2026; Han et al., 2026). When the goal is to learn an unknown mechanism or diagnose identifiability, tight coupling is uniquely suited (Bisht and Agarwal, 2026). And when the model must survive regulatory scrutiny, mechanism-grounded outputs from medium or tight coupling should be preferred over black-box learners (Lu et al., 2021b; Raissi et al., 2019; Valderrama et al., 2024; Tsiros et al., 2026).

**FIGURE 8:**
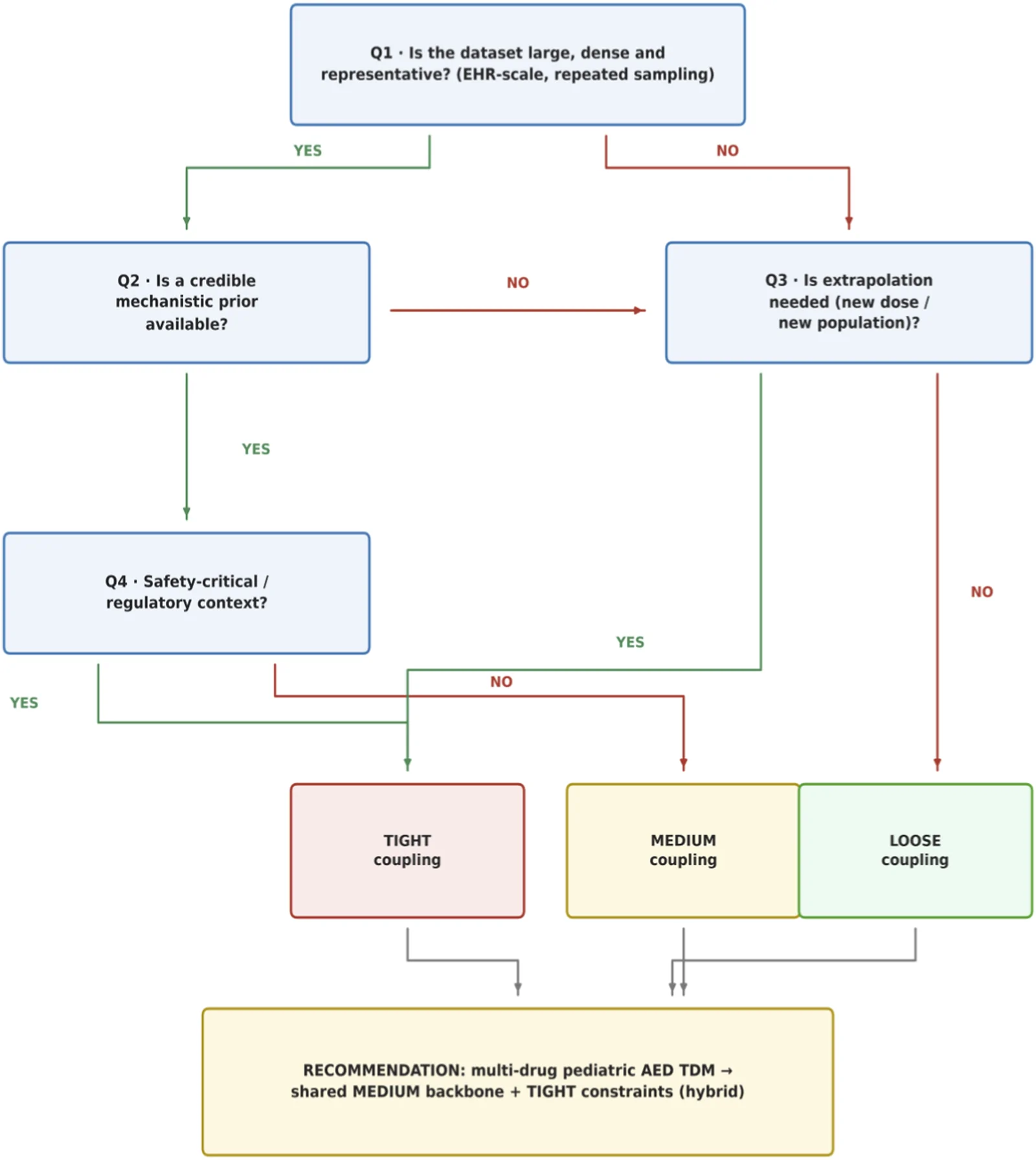
Decision guide for choosing the coupling depth (Section 5.5). Four questions, on data scale (Q1), mechanistic prior availability (Q2), task type (Q3), and regulatory/clinical context (Q4), lead to loose, medium, or tight coupling, with a hybrid design recommended for portfolio settings such as multi-drug pediatric AED TDM.

The pediatric antiepileptic drug (AED) TDM setting that motivates this review shows how the decision plays out in practice. New-generation AEDs such as lamotrigine, oxcarbazepine, and lacosamide have narrow therapeutic windows and sparse, developmentally heterogeneous sampling; their PopPK models cover only a subset of covariates, and multi-drug generalization remains open (Chen J. et al., 2025). A loose-coupled PPK + ML model, as built for valproic acid (Chen J. et al., 2025) and for single drugs in China (Chen et al., 2026; Liu R. et al., 2025; Zhang et al., 2025), is a sensible first step, though it inherits the limitations of Section 5.1. A medium-coupled neural-PK/PD or SciML architecture would exploit the known compartmental structure of AED disposition while learning drug-specific kinetics (Lu et al., 2021b; Valderrama et al., 2024). A tight-coupled PINN-style model with distributional outputs (Tsiros et al., 2026) could enforce mass-balance constraints and quantify uncertainty in exactly the data-scarce regimes where AED dosing decisions matter most. The practical answer is a portfolio, with a shared medium-coupled backbone, tight-coupling constraints for the most safety-critical drugs, and loose-coupled modules where rapid deployment matters. Two further considerations follow from real-world evidence. First, temporal information (the patient’s own prior concentration history) consistently dominates static covariates in head-to-head comparisons on the same cohort, so any pediatric AED system should privilege sequence-aware architectures regardless of coupling depth (Chen J. et al., 2025; Ghanbari et al., 2026). Second, the attainable accuracy is capped by the information ceiling of the sampling design (Section 8.1); breaking it requires denser longitudinal sampling and routine capture of renal function and sampling-type metadata alongside better models, a point we return to in Section 8. The framing bears repeating. Kids are not small adults, dosing them is a high-stakes game, mechanism is the safety net, and sequence information beats static covariates.

## 6 Quantitative cross-study synthesis and critical appraisal

The taxonomy of Section 5 is only useful if the underlying evidence is interpreted with appropriate caution. In this section we (i) harmonize the performance comparisons reported by the included studies into a single cross-study view (Figure 4), (ii) provide a bibliometric and citation analysis of the two research streams (Figure 5), and (iii) critically appraise the statistical quality of the key primary-study results, applying to the literature the standards we recommend to the field.

Before harmonizing numeric results, we locate each included study on the evidence hierarchy that separates concentration prediction from clinically consequential dosing. Figure 9 defines five levels: (1) concentration prediction, (2) pharmacokinetic/exposure-parameter estimation, (3) dose recommendation, (4) clinical decision support, and (5) prospective clinical-outcome improvement. Almost all included studies operate at levels 1–2; a handful reach level 3 (e.g., Nephrocast-V (Ghanbari et al., 2026), which issues a dose recommendation together with a predicted attainment probability); level 4, integration into routine workflow, is at best retrospective; and no included study demonstrates level 5. The hierarchy matters because predictive accuracy at lower levels does not automatically translate into better dosing decisions or improved patient outcomes: a more accurate concentration forecast still leaves the dose-selection policy, the clinical workflow, and the outcome measure unaddressed. We therefore interpret every performance claim in Sections 6.1–6.3 through this lens (Figure 9).

**FIGURE 9:**
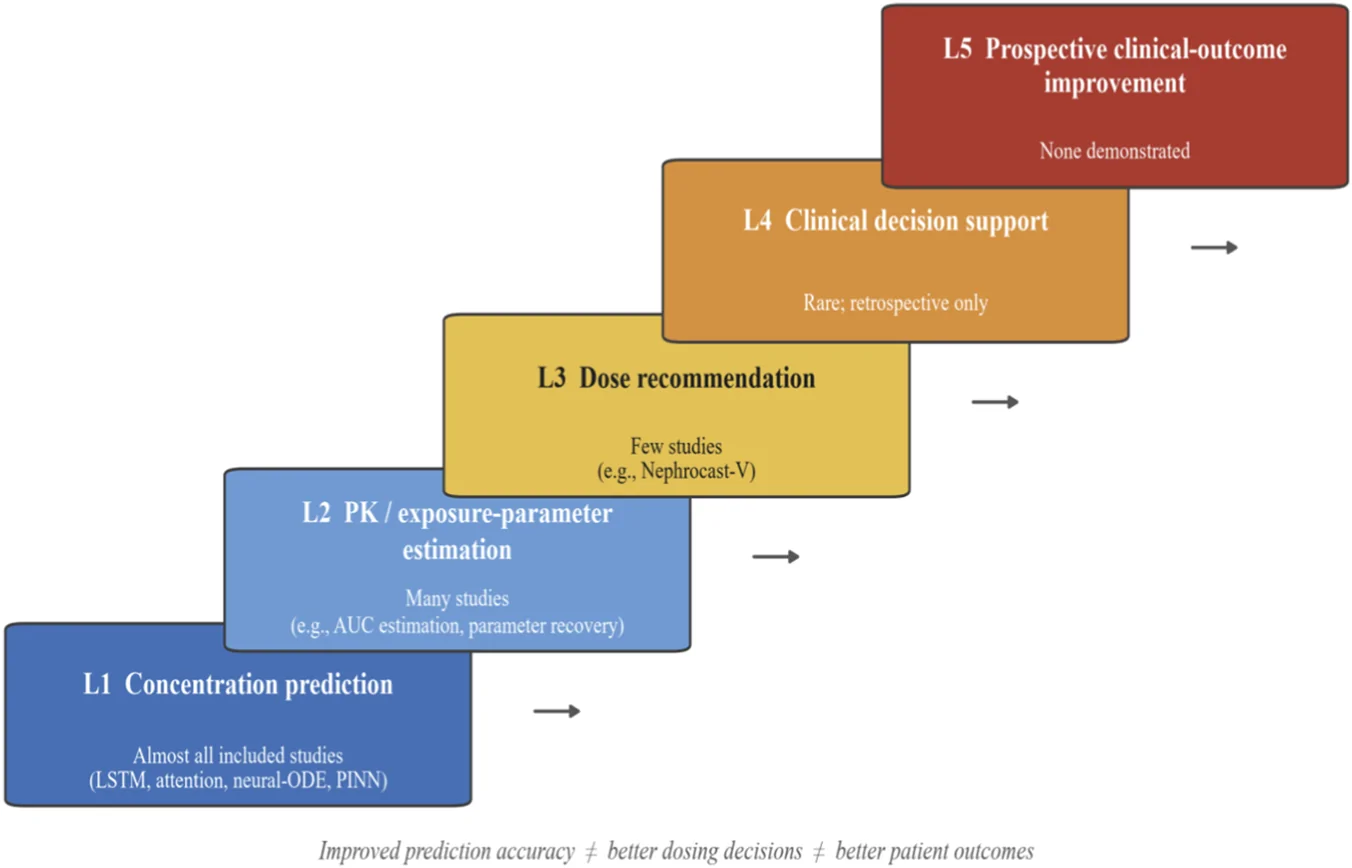
Evidence hierarchy for AI-based dosing research. Levels ascend from concentration prediction (L1) to prospective clinical-outcome improvement (L5). For each level we indicate the status of the evidence reviewed here: almost all included studies operate at L1-L2; dose recommendation (L3) is reached by few studies; clinical decision support (L4) is rare and retrospective; and no included study demonstrates prospective outcome improvement (L5). Predictive accuracy at lower levels does not by itself constitute precision dosing.

### 6.1 Harmonized cross-study comparison

Table 2 and the studies reviewed above report a heterogeneous set of metrics (RMSE, MAE, MAPE, F20/F30, R^2^) measured on different drugs, populations, and validation designs, so no pooled effect size is statistically meaningful. We therefore harmonize only the comparison against each study’s stated comparator, expressing the result as the relative difference in the reported performance metric between the proposed model and the comparator (negative values favor the proposed model; Figure 4). Four estimable comparisons emerge. Khusial et al. report an RMSE of 29.566 versus 31.129 for NONMEM PopPK, a 5.0% improvement (Khusial et al., 2023). Nephrocast-V reports therapeutic-trough attainment of 55.6% versus 56.7% for a critical-care pharmacist, a 1.9% deficit that was not statistically significant (p = 0.24) (Ghanbari et al., 2026). Sim2Real-PK reports F50 gains from 32.1% to 39.9% for ibrutinib (24.3% relative improvement) and F20 gains from 38.8% to 51.1% for daratumumab (31.7% relative improvement) on external YODA individual-patient data (Han et al., 2026). Zhang et al.'s R^2^ of 0.9993 is excluded from the display because the test set was simulated from the same PopPK model used to generate the training signal; that number is circular, not external, and is flagged in Table 2 and Section 6.3 (Zhang et al., 2025). Two further studies report superiority without extractable numeric deltas (the valproic acid NN with F30 above 80% (Chen J. et al., 2025); the SciML extrapolation results of Valderrama et al. (2024)), and the D-PINN reports accuracy comparable to MCMC on aggregated data without a delta (Tsiros et al., 2026).

Three observations follow directly from Figure 4. First, of the four estimable comparisons, only two are both peer-reviewed and validated on real clinical data (Khusial et al., 2023 and Nephrocast-V Ghanbari et al., 2026), and their effects are small (−5.0% and +1.9%, neither with confidence intervals). Second, the largest reported effects come from a preprint (Sim2Real-PK (Han et al., 2026)) validated on external trial data; its magnitude, if confirmed by peer review, is precisely the kind of evidence the field needs. Third, no study in the included set reported a confidence interval or significance test for its headline performance delta. The “weight of evidence” in this review is therefore best summarized as follows: mechanism-constrained and sequence-aware models show consistent, credible advantages in extrapolation and identifiability tasks, while their in-distribution accuracy advantages over well-tuned PopPK + MAPB are, at present, modest, heterogeneous, and largely unreplicated. We deliberately refrain from declaring a global winner and instead provide the decision guide of Section 5.5.

### 6.2 Bibliometric context and cross-citation analysis

The claim that the two research streams “rarely cite each other” should rest on data rather than impression. PubMed-indexed publication counts for the two primary queries (Section 2) by year are shown in Figure 5. Stream A grew from 27 records in 2019 to 253 in 2025 and 283 in the partial year 2026, an approximately tenfold increase, consistent with the field’s shift from proof-of-concept to EHR-scale deployment. Stream B, by contrast, remained essentially invisible in PubMed during 2019–2022 (0 records), appeared in 2023 (n = 1), and reached only 4 (2024), 8 (2025) and 10 (2026, partial) records. This near-absence in PubMed does not mean the physics-constrained stream is inactive: most of its output circulates on preprint servers (arXiv, Research Square) and in proceedings, which PubMed undercounts, a point that reinforces the evidence-stratification argument of Section 6.1.

Cross-citation between the two streams was assessed by inspecting the reference lists of the included primary studies. We verified the two time-series cornerstone studies directly: the reference list of Khusial et al. (2023) contains 51 entries, none of which cites the PINN/SciML literature; the reference list of Lu et al. (2021a) contains 37 entries, also with no PINN/SciML citations (the historical anchor “Forecasting individual pharmacokinetics” (Sheiner et al., 1979) does appear, confirming that the stream is rooted in Bayesian forecasting rather than in physics-constrained learning). Conversely, the physics-constrained preprints (Bisht and Agarwal, 2026; Liu S. et al., 2025; Liu et al., 2026; Han et al., 2026) cite sequence models mainly as generic baselines, not as the TDM literature reviewed here; only Valderrama et al. (2024), Tsiros et al. (2026) engage substantively with the machine-learning literature. The two communities thus operate with near-zero mutual citation, exactly the gap that the coupling taxonomy and the present synthesis are intended to bridge. To keep this analysis current, the full per-study citation-link log is maintained in the Supplementary Material.

### 6.3 Critical appraisal of primary-study statistics

We apply to the primary literature the same appraisal standards we recommend to the field, focusing on three cases.

First, the R^2^ = 0.9993 reported on the test set of the vancomycin AUC24h model of Zhang et al. (2025) is not interpretable as generalizable accuracy. The XGBoost model was trained on 4,000 concentration-time curves simulated from a fitted PopPK model, and the validation set was drawn from the same simulation machinery. Training and evaluating on curves generated from the same structural model means the test set shares the model’s parametric assumptions and noise structure; the near-perfect R^2^ largely reflects how faithfully the surrogate reproduced the simulator, not how well either model predicts a patient’s true AUC24h. We flag this as circularity (Table 2) and exclude the value from the harmonized comparison of Figure 4. The clinically meaningful result in that study is the head-to-head against the Bayesian estimator on real patient data, which should be reported with its own uncertainty.

Second, the olanzapine comparison of Khusial et al. (2023) reports an RMSE difference of 29.566 versus 31.129 (about 5%) without confidence intervals, significance testing, or repeated-split variability. On 1,527 observations from 523 individuals, a 5% RMSE delta can easily lie within sampling variability, particularly for a metric as noisy as RMSE on sparse trough data. We therefore treat this as weak evidence of superiority and recommend that head-to-head model comparisons report bootstrap or repeated cross-validation confidence intervals as a reporting standard (Section 8.2).

Third, the Nephrocast-V comparison with the critical-care pharmacist (Ghanbari et al., 2026) was reported as non-significant (p = 0.24) with respect to therapeutic-trough attainment (55.6% vs. 56.7%), not with respect to MAE. Two corrections follow. The original Figure 7b plotted MAE against the pharmacist, implying a comparison that was never reported; we have redrawn the panel using the attainment metric (Figure 7b). More substantively, no non-inferiority margin was pre-specified, so the non-significant difference cannot be read as equivalence; a p-value above 0.05 with a 1.9-point deficit in attainment is simply an underpowered result. Table 2 now records this caveat, and Section 8.4 argues that prospective, pre-specified, margin-based designs are the path to regulatory-grade evidence.

## 7 Open-source ecosystem and reproducibility

A distinctive strength of this field is its open ecosystem, which spans datasets, modeling platforms, and method-specific codebases. At the data layer, the Medical Information Mart for Intensive Care IV (MIMIC-IV) is the anchor, a freely accessible, single-center EHR database covering more than 73,000 ICU admissions at the Beth Israel Deaconess Medical Center (2008–2019), released on PhysioNet under a data-use agreement (Johnson et al., 2023). MIMIC-IV has enabled both the EHR-scale Nephrocast-V application (Ghanbari et al., 2026) and a family of vancomycin-related analyses, from TDM outcome studies to machine learning pipelines for dose prediction, and it remains the *de facto* benchmark for AI-TDM research. Complementary resources include Therapeutics Data Commons (TDC), an AI-ready benchmark suite with ADME/TDM-related tasks, and the aggregated concentration data that D-PINNs are specifically designed to exploit (Tsiros et al., 2026; Therapeutics Data Commons, 2026). The pattern is worth stating plainly. Openness compounds, shared assets raise everyone, and the pipeline stays public.

At the platform layer, the mechanistic side is exceptionally well served. nlmixr2, an open-source R package for NLME modeling, provides a natural source of PopPK priors for the coupling approaches of Section 5. The Open Systems Pharmacology (OSP) Suite (PK-Sim and MoBi) is a GPL-licensed, whole-body PBPK/QSP platform. It ships human and preclinical physiology databases, supports pediatric extrapolation workflows, offers an R/Excel interface, and its Qualification Framework has been used for regulatory submissions. Its community and qualification reports are themselves open (Lippert et al., 2019). The drug-discovery side contributes the ATOM Modeling PipeLine (AMPL), an end-to-end pipeline for curating data, building models, and predicting potency, safety, and pharmacokinetic-relevant parameters (ATOM Modeling PipeLine, 2026), and MMPK, a multimodal deep learning model that predicts eight human oral pharmacokinetic parameters from molecular structure, with a public web server and downloadable pretrained weights (MMPK, 2026). The tooling is mature, the mechanistic platforms are free, and adoption is close to frictionless.

The commercial MIPD landscape deserves explicit treatment, because the claim that PopPK + MAPB “sits inside most commercial TDM software” requires naming names. Systematic evaluations of MIPD software tools identified ten to nineteen platforms, depending on the review (Kantasiripitak et al., 2020; Del Valle-Moreno et al., 2023): on the commercial side, InsightRX Nova, DoseMeRx, MwPharm++ and PrecisePK; on the non-commercial side, AutoKinetics, BestDose, ID-ODS, NextDose, TDMx, Tucuxi and others (Kantasiripitak et al., 2020; Del Valle-Moreno et al., 2023; Fuchs et al., 2013). All of these platforms are built on NLME PopPK model libraries with Bayesian maximum *a posteriori* estimation as their engine; to the best of our knowledge, as of the search date, none ships a deployed deep-learning or physics-constrained module, and only a few offer the pediatric or obstetric model coverage relevant to this review’s motivating scenario (Kantasiripitak et al., 2020; Del Valle-Moreno et al., 2023). This is a gap and an opportunity simultaneously: a mechanism-constrained predictor that preserves the Bayesian workflow clinicians already use could be integrated into existing MIPD platforms as a module, reusing their model libraries, validation habits, and EHR interfaces, rather than competing with them, an argument we return to in Section 8.4.

At the method-code layer, both research lines of this review are directly reproducible from public repositories. For time-series and neural-ODE modeling, pharmacoNODE provides a clean Python implementation of neural-ODE pharmacokinetics compatible with Monolix/NONMEM data formats; it was applied to dalbavancin in 218 patients and accompanied by a peer-reviewed methodology paper in Entropy (Giacometti et al., 2025; Giacometti, 2026). The vancomycin_dose_predictions pipeline ships MIMIC-IV extraction SQL plus LSTM and gradient-boosting training code with nested cross-validation and dose-response simulation (O'Callaghan, 2026). The SCAI-Lab MIMIC-IV pipeline offers a full extract-process-train-evaluate workflow for EHR modeling (SCAI-Lab MIMIC-IV Data Pipeline, 2026). For physics-constrained modeling, the PINN lineage is fully open from its origins (Raissi et al., 2019), and the D-PINN, chemotherapy-PINN, and physiologically informed frameworks reviewed in Section 4 have public preprint code or are associated with reproducible implementations (Tsiros et al., 2026; Bisht and Agarwal, 2026; Liu et al., 2026). Reproducibility is the quiet standard here. Code is evidence, and open ecosystems compound.

Together these resources make the full pipeline, from data curation and mechanistic prior construction through time-series modeling to mechanism-constrained training and decision support, reproducible at near-zero cost, a major enabler of the hybrid approaches reviewed here. Two caveats deserve mention. The first concerns data access. Several key clinical datasets, such as dalbavancin in pharmacoNODE and some YODA-derived analyses, are available only on request or under license, limiting direct replication (Giacometti, 2026). Preprint codebases can also drift from the published version of record, so we recommend that method papers release versioned, archived code (e.g., on Zenodo) alongside the manuscript, mirroring the practice of the OSP community (Lippert et al., 2019). The balance sheet is simple. Open data anchor the field, open code carries the method, and both are assets worth protecting.

## 8 Challenges, opportunities and future directions

### 8.1 Data scarcity, sparsity and heterogeneity

The single most binding constraint on deep learning in pharmacometrics is data. Pharmacokinetic data are inherently sparse, typically a handful of samples per patient, often trough-only, and irregularly sampled. They combine measurement noise, inter-occasion variability, and clinical confounding; and because they are produced by routine care, they carry missing values, dosing irregularities, and indication bias (Khusial et al., 2023; Chen J. et al., 2025). Deep learning thrives on scale and density, yet the largest public EHR datasets relevant to TDM, such as MIMIC-IV with over 73,000 ICU admissions (Johnson et al., 2023), yield only a few thousand usable concentration records per drug (Ghanbari et al., 2026). The consequence is that most published models are trained on a few hundred patients, single-center, with internal validation only, and performance on external cohorts frequently degrades (Khusial et al., 2023; Chen J. et al., 2025). A useful way to frame this scarcity is through the concept of an information ceiling, the idea that the sampling design itself fixes an upper bound on the accuracy any model can achieve, regardless of architecture or coupling depth. When a large fraction of patients contribute only a single trough measurement, and when the within-patient coefficient of variation of repeated troughs approaches the ±30% tolerance used to judge predictive performance, even an oracle model cannot exceed the F30 set by that noise. Honest baselines, predicting the patient’s previous concentration or the patient-specific mean, quantify this ceiling and separate data-limited from model-limited performance. Recent real-world head-to-head studies illustrate the point. Temporal deep learning outperformed both PopPK + MAPB and cross-sectional ML on the same cohort, yet all approaches plateaued below a clinically actionable F30 of about 0.80, not because the models were weak but because single-timepoint, covariate-incomplete sampling did not contain the information to do better (Khusial et al., 2023; Ghanbari et al., 2026). Recognizing this ceiling redirects effort toward the two levers that actually move it, denser longitudinal sampling (which adds temporal information) and mechanism constraints (which supply structure where measurements are absent), the joint agenda of Sections 5 and 8.3. The information ceiling is structural; denser sampling is the only escape, and mechanism helps where data end.

Several mitigation strategies are already visible in the literature, and we believe their systematic combination is the field’s most important near-term opportunity. One is synthetic-data pretraining. Sim2Real-PK pretrains a neural dynamics core on massive mechanistic ODE-generated datasets before adapting to real clinical data (Han et al., 2026), and the vancomycin AUC24h study of Zhang et al. trained its XGBoost model on 4,000 PopPK-simulated curves (Zhang et al., 2025), both showing that mechanism-based simulation can make up for scarce real data. Another is the reuse of aggregated literature data. D-PINNs recover population parameter distributions from published means and variances alone, unlocking decades of summary-level PK literature that individual-level analyses cannot access (Tsiros et al., 2026). A third is data augmentation tailored to PK structure, such as the observation-window truncation used in early neural-ODE work (Lu et al., 2021a). Beyond these, federated learning across institutions (already prototyped in EHR research) and the development of PK-specific benchmark suites built on harmonized multi-center cohorts would raise the evidence bar for the entire field. Mitigation exists, the toolbox is ready, and combination is the strategy. The levers are known, and the will is the question.

### 8.2 Identifiability and uncertainty quantification

Mechanism-constrained models surface identifiability problems rather than hiding them. We regard that as a feature, but it converts a technical issue into a scientific obligation. If a model cannot distinguish competing explanations from the data it has, then its predictions in the non-identifiable region should carry the corresponding uncertainty, not a false precision (Bisht and Agarwal, 2026). Bisht and Agarwal’s demonstration is a template for how identifiability analysis should be reported in pharmacokinetic deep learning (Bisht and Agarwal, 2026). Plasma-only data render the Michaelis-Menten two-compartment model structurally non-identifiable, and sparse tissue observations largely restore identifiability. The takeaway is straightforward. Point estimates hide doubt, intervals show it, and calibration is the goal.

Uncertainty quantification (UQ) remains underdeveloped across all three coupling stages. Point predictions dominate the literature; interval estimates, predictive distributions, and calibrated confidence are rare. The available toolbox is substantial. D-PINN’s distributional output provides a principled, mechanism-consistent probabilistic framework (Tsiros et al., 2026); Bayesian PINNs and deep ensembles can quantify parameter and prediction uncertainty within the tight-coupling setting; conformal prediction, which constructs prediction intervals with finite-sample coverage guarantees from held-out residuals and requires no distributional assumptions about the model or the data, offers a model-agnostic route to calibrated uncertainty on top of any of the three coupling stages; and posterior predictive checks can validate whether mechanistic constraints are consistent with the data. Calibration is the step that makes uncertainty clinically usable. An interval is only trustworthy if a nominal 90% interval actually contains the observed concentration about 90% of the time, and none of the studies reviewed here reports explicit calibration checks such as empirical coverage or probability integral transform diagnostics. Whether coupling depth influences calibration, for example, whether tight coupling yields better-calibrated uncertainty than loose or medium coupling by anchoring predictions in mechanism, is an open question that deserves explicit investigation. We encourage the community to adopt at least one of these as a reporting standard, and to report calibration alongside coverage, because dose decisions are inherently decisions under uncertainty. Clinicians need to know how much to trust a recommendation, not just what the recommendation says. The question is always the same. How much should the dose change, and how sure are we? If the data cannot tell the models apart, we should say so. Uncertainty is the honest answer.

### 8.3 Multi-drug generalization and special populations

Most of the models reviewed here are single-drug and single-center (Chen et al., 2026; Liu R. et al., 2025; Zhang et al., 2025; Liu S. et al., 2025). That is a natural first step but a strategic limitation. TDM services manage dozens of drugs with shared pharmacokinetic structure, and per-drug bespoke models are neither scalable nor generalizable. Cross-drug generalization, transferring knowledge from well-characterized drugs to under-characterized ones, is a largely open problem, and the mechanistic priors that medium and tight coupling provide are precisely the kind of shared inductive bias that could enable it. Foundation-style pretraining on multi-drug PK corpora (Liu et al., 2026) is one direction; pharmacologically structured multi-task learning, in which the shared compartmental and developmental structure of AEDs is encoded as a common inductive prior across lacosamide, lamotrigine, and oxcarbazepine, is another and arguably the more immediate route, since it builds on the established PopPK models of each drug rather than requiring pretraining corpora that do not yet exist. One drug at a time is not enough. Shared structure is the key, and generalization must be engineered.

Special populations, and pediatrics in particular, concentrate all of these difficulties. Developmental pharmacokinetics change body composition, enzyme maturation, and clearance continuously, so adult models cannot be transferred directly. Pediatric sampling is even sparser than adult sampling, for ethical and practical reasons, and narrow therapeutic windows make dose errors consequential. The pediatric AED TDM gap identified in Section 3.4, where new-generation AEDs (lamotrigine, oxcarbazepine, lacosamide) lack integrated time-series-plus-mechanism-constrained decision tools in contrast to the well-studied valproic acid (Chen J. et al., 2025), is a concrete, high-value target. The decision framework of Section 5.5 argues that a shared medium-coupled backbone with tight-coupling constraints is the most promising architecture for such multi-drug pediatric systems. A realistic near-term blueprint is a multi-drug pediatric AED platform trained jointly across lacosamide, lamotrigine, and oxcarbazepine, exploiting shared pharmacokinetic structure and co-medication effects, with tight-coupling constraints reserved for the most safety-critical drugs and with the information ceiling of Section 8.1 used to set honest performance targets. Such a platform would directly address the multi-drug, sequential-therapy reality of pediatric epilepsy management. The situation is unforgiving in its own way. Kids metabolize differently, samples are scarce, and mechanism is what fills the gap.

Developmental pharmacology offers the clearest case for mechanism-constrained modeling, because the mechanism is partly known and partly developmental. Pediatric clearance changes with body size and with the maturation of specific pathways—CYP3A4/1A2 for many AEDs, UGT1A4-mediated glucuronidation for lamotrigine, renal function for renally cleared drugs—and these changes are routinely captured in pharmacometrics by allometric scaling and by ontogeny (maturation) functions (Anderson and Holford, 2008; Anderson and Holford, 2013; Kearns et al., 2003). In the coupling framework of Section 5, this prior knowledge enters naturally as a mechanistic prior: allometric exponents with freely estimated values (as in the lacosamide pediatric model, where the CL exponent was estimated rather than fixed (Winkler et al., 2019)), maturation functions for enzymes of known ontogeny (Anderson and Holford, 2008; Anderson and Holford, 2013), and developmental covariates inside the physics loss of tight-coupled models (Tsiros et al., 2026; Bisht and Agarwal, 2026). Two concrete connections follow. First, the “Neural Allometry” component of the physiologically informed framework (Liu et al., 2026), which learns continuous cross-species scaling laws, is directly relevant to pediatric extrapolation workflows already embedded in platforms such as PK-Sim (Section 7): a learned allometry could be calibrated on adult data and constrained to reproduce known pediatric maturation, replacing purely empirical allometric priors (Lippert et al., 2019; Anderson and Holford, 2008). Second, for the pediatric AED scenario that motivates this review, the existing PopPK literature already supplies the developmental priors: lamotrigine clearance in Chinese children is described by one-compartment models with body weight and co-medication covariates (He et al., 2012); oxcarbazepine’s active metabolite MHD has been modeled in Chinese children with age, weight and daily-dose-per-weight covariates (Peng et al., 2014) and in European children with parent-metabolite structure and allometric scaling (Rodrigues et al., 2017); lacosamide clearance scales allometrically with body weight and is reduced by enzyme-inducing AEDs (Winkler et al., 2019). These models are exactly the mechanistic anchors that a multi-drug pediatric AED platform (Section 8.3) would need, and Table 4 maps what exists, what is missing, and where the gaps are.

**TABLE 4:** Evidence gaps for new-generation pediatric antiepileptic drugs, mapped against the well-studied reference drug valproic acid.

Drug	Existing pediatric PopPK models (covariates; age range; method)	Existing ML/hybrid models	Typical sampling density (TDM practice)	Identified gaps
Valproic acid (reference)	PopPK models with external validation; multi-model comparison including NN (Chen et al., 2025a)	NN, RF/XGBoost/LightGBM, MLR, MAPB with F30 > 80%, externally validated (Chen et al., 2025a)	Routine troughs; multi-center archives	No mechanism-constrained tool; temporal information underused
Lamotrigine	One-compartment PPK in Chinese children (NONMEM): CL scaled by TBW with VPA/CBZ/PB covariates; n = 284 (He et al., 2012). Validation of adjunctive-therapy models in children available	None identified in the searched literature	Sparse troughs (1–3 per patient); TDM-driven	No ML/hybrid model; *UGT1A4*/*UGT2B7* genotype never integrated (Petrenaite et al., 2022); no time-series or mechanism-constrained model; developmental maturation not explicitly modeled
Oxcarbazepine (active metabolite MHD)	MHD PPK in Chinese children (Phoenix NLME): age, TBW, dose-per-weight, gender; n = 466 (Peng et al., 2014). Parent–metabolite model in European children (Monolix): allometric, EIAED covariate; n = 31 (Rodrigues et al., 2017)	​	Sparse MHD troughs	Enantiomer-specific PK (S/R-MHD) not modeled; few external validations; no mechanism-constrained model; pediatric ML reports unverified
Lacosamide	Allometric pediatric PopPK (NONMEM): freely estimated CL exponent, EIAED covariate; 6 months–18 years; n = 79 trial patients (Winkler et al., 2019); external validation in Chinese children with MAPB, n = 64 (Chen et al., 2025b)	None identified	Sparse troughs; TDM not yet routine in China	No Chinese pediatric model built from local data (external validation only) (Chen et al., 2025b); no ML/hybrid model; *CYP2C19* effect minor per label but untested in dosing tools; no mechanism-constrained model

Pediatric AED, evidence-gap analysis for the guiding clinical scenario of this review. PopPK, population pharmacokinetics; PPK, population pharmacokinetics (Chinese nomenclature); ML, machine learning; MAPB, maximum *a posteriori* Bayesian estimation; MHD, 10-monohydroxy derivative of oxcarbazepine; EIAED, enzyme-inducing antiepileptic drug; TBW, total body weight; VPA, valproic acid; CBZ, carbamazepine; PB, phenobarbital; NN, neural network; NR, not reported. The table converts the motivational framing of the Introduction into a systematic, study-level map: for each drug we list what exists (PopPK, models with covariates and age ranges), what ML/hybrid capacity exists, the sampling density that constrains all approaches (information ceiling, Section 8.1), and the specific gaps that a multi-drug, mechanism-constrained platform would fill (Section 8.3).

Pregnancy is the second special population where mechanism-first thinking is not a luxury but the only ethical option, because randomized PK studies in pregnant women are largely infeasible. Pregnancy alters drug disposition profoundly—increased glomerular filtration and renal blood flow, induction of *UGT1A4* and CYP3A4, reduced CYP1A2—and these changes are captured in physiology-based models rather than by extrapolating adult data (Dallmann et al., 2018). The AED example is instructive: lamotrigine clearance rises markedly during pregnancy as *UGT1A4* is induced, so dose requirements increase and fall again postpartum, a pattern documented by prospective TDM studies (Pennell et al., 2008) and by UGT pharmacogenetics work (Petrenaite et al., 2022). Physiologically based models for pregnant women have been qualified against observed PK for CYP- and renal-cleared drugs and are now used to support dose decisions before any *in-vivo* pregnancy data exist (Dallmann et al., 2018). Within the framework of this review, pregnancy PBPK is a mature instance of medium-to-tight coupling: fixed physiological structure, known gestation-specific enzyme trajectories, and learned or estimated drug-specific parameters. It provides a template, and a regulatory precedent, for how mechanism-constrained models can serve populations in which prospective data are unobtainable, which is precisely the argument that carries over to pediatric AED TDM. We therefore regard the obstetric and pediatric pharmacology sections of this journal as sharing a single methodological agenda: replace extrapolation from adults by explicit, mechanism-constrained developmental and gestational priors.

### 8.4 Clinical and regulatory acceptance

The principal barrier to clinical and regulatory acceptance of AI in dosing is the black-box problem. Clinicians and regulators cannot act on recommendations whose provenance and mechanism they cannot inspect. Mechanism-constrained models directly address this concern, because their outputs are anchored to pharmacokinetic laws and their parameters retain physiological meaning (Lu et al., 2021b; Raissi et al., 2019; Valderrama et al., 2024; Tsiros et al., 2026; Liu et al., 2026). Early evidence suggests that clinicians respond positively to models that can explain their outputs in pharmacokinetic terms. Nephrocast-V, for instance, performed comparably to a critical-care pharmacist aided by Bayesian software, and dose-recommendation deviations correlated with non-therapeutic troughs (Ghanbari et al., 2026). Nevertheless, acceptance will ultimately rest on evidence quality, including prospective validation, decision-support integration (embedding in EHR and CDSS workflows), and standardized reporting. We recommend that ML-TDM studies adopt established reporting frameworks (e.g., TRIPOD-AI/DECIDE-AI analogues), pre-register analyses, and publish code and data where permissible, mirroring the transparency now expected of classical pharmacometric analyses. Regulators need to see inside; mechanism helps them do so, and reporting standards should follow.

From a regulatory perspective, model-informed drug development (MIDD) is already accepted by agencies including the FDA and EMA for PopPK and PBPK modeling, and the incremental step is to extend that acceptance to mechanism-informed machine learning. Both agencies have begun to specify how AI will be handled. The FDA’s draft guidance proposes a risk-based credibility assessment framework organized around context of use (US Food and Drug Administration, 2025), and the EMA’s reflection paper on AI in the medicinal product lifecycle sets expectations for data, validation, interpretability, and governance across the lifecycle (European Medicines Agency, 2024). Model credibility, uncertainty quantification, and validation discipline are becoming the explicit currency of regulatory acceptance, which is exactly the direction in which medium- and tight-coupled models already point. The qualification infrastructure of the Open Systems Pharmacology community (Lippert et al., 2019), with its documented, validated, community-reviewed model repositories, offers a template for how mechanism-constrained ML models could be qualified for regulatory use. We believe that the combination of mechanistic consistency, formal UQ, and transparent reporting positions tight- and medium-coupled models to gain regulatory acceptance far faster than black-box predictors, provided the community commits to those standards now. The path is clear even if the destination is years away. Prospective trials settle it, retrospective data do not, and the evidence bar is high.

A decisive and still missing step is the transition from retrospective analysis to prospective, interventional validation. Nearly all models reviewed here, including Nephrocast-V (Ghanbari et al., 2026) and the Chinese single-center studies (Chen et al., 2026; Liu R. et al., 2025; Zhang et al., 2025), were developed and evaluated on retrospective EHR or TDM data. That establishes predictive accuracy; it does not establish clinical effectiveness. Demonstrating that AI-driven dose-recommendation systems actually improve patient outcomes, through higher rates of therapeutic-range attainment, fewer concentration-related toxicities, or reduced time to target exposure, for example, requires randomized or at least prospectively embedded study designs in which model recommendations are compared against standard-of-care dosing. Analogous evidence exists in adjacent fields, such as clinical decision support in critical care, but not yet for pharmacometric AI. We regard such prospective, intervention-focused trials as the single most important determinant of whether the approaches reviewed here enter routine practice, and we encourage study teams and funders to prioritize them alongside methodological development. The endpoint of such validation should be a closed decision loop, monitoring, prediction, dose recommendation, and outcome feedback, rather than a standalone concentration predictor. Embedding mechanism-constrained models in EHR and CDSS workflows, where the model’s recommendation and its pharmacokinetic rationale are both visible to the clinician, is the concrete form this transition takes; pediatric AED TDM, with its clear endpoints of seizure control and concentration-related toxicity, offers one of the most tractable settings in which to establish that evidence.

### 8.5 Foundation models and the future of the field

The most recent developments reviewed here signal a shift toward scale, with Foundation PBPK Transformers that treat pharmacokinetic forecasting as sequence modeling, Physiologically Constrained Diffusion Models (PCDM) for generating biologically compliant virtual patient populations, and Neural Allometry for continuous cross-species scaling (Liu et al., 2026). A foundation model for pharmacology would be pretrained on large, multi-source corpora, including EHRs, clinical trials, literature summaries, and mechanistic simulations, then fine-tuned per drug, per population, or per clinical site, with physiological constraints retained through the tight-coupling machinery of Section 5.3. If realized, such a model could unify the two lines this review surveys. Time-series deep learning contributes representational capacity and sequence modeling, while physics-constrained modeling contributes mechanism, consistency, and trust. The caution is obvious. Large models bring large risks, mechanism keeps them honest, and the path is narrow.

What could foundation models deliver here? Virtual patients for clinical trial design (Liu et al., 2026), extrapolation across species and populations, zero-shot dose predictions for drugs with no clinical data at all, and continuous *in silico* dose titration. The risks deserve equal attention. Training corpora carry biases, and a foundation model will inherit and amplify them. If the pretraining data over-represent adults, or certain ethnicities or body compositions, fine-tuning propagates those biases into pediatric or under-represented populations, with real dosing consequences. For PK models, the biases most likely to matter are demographic in nature. Body weight and body-surface area drive clearance and volume scaling for most drugs, and organ-function covariates such as renal and hepatic function differ systematically across ancestry and age, so a corpus skewed along any of these axes will silently skew the doses it recommends. Catastrophic forgetting is a second, less recognized hazard. Fine-tuning a large pretrained model on a small single-drug TDM cohort can overwrite the general pharmacokinetic knowledge acquired in pretraining, silently degrading performance on drugs or populations missing from the fine-tuning data. Low-rank adaptation, rehearsal of synthetic mechanistic data, and regularization against the pretrained weights are plausible mitigation strategies, but PK-specific evaluations of fine-tuning strategies are lacking, and whether these techniques preserve pretrained pharmacokinetic knowledge across drugs and populations remains an open research question. Beyond these technical risks, weakened physics constraints could let foundation models hallucinate plausible but wrong dynamics; their computational footprint exceeds what most hospital analytics teams can sustain; and their outputs remain subject to the identifiability and UQ concerns of Section 8.2. Ethical and fairness dimensions, including equitable performance across demographic groups, transparent failure modes, and human oversight of automated dose changes, need to be designed in from the start, not retrofitted. Mechanism-informed, foundation-scale, rigorously validated precision-dosing systems are now technically plausible; whether they reach the bedside or stay in the laboratory (Figure 3) will depend on the community’s choices over the next few years, especially around validation standards and open science. Pediatric AED TDM, where data are scarcest and stakes are highest, is among the most promising proving grounds, and the multi-drug platform outlined in Section 8.3, new-generation AEDs trained jointly under mechanism constraints, is the natural first candidate for foundation-model fine-tuning (Chen J. et al., 2025).

## 9 Conclusion

What began as two separate curiosities, time-series deep learning and physics-constrained PopPK modeling, has become a converging and rapidly maturing field. Across the studies published between 2019 and 2026, we observe a steady movement toward deeper mechanistic coupling, from pharmacokinetic-derived features fed into machine learning (loose), to mechanism-embedded architectures (medium), to differentiable mechanism constraints in the training objective (tight). The pattern repeats across representative studies, where mechanism constraints improve extrapolation and interpretability without sacrificing accuracy, although, as the stratified evidence map (Table 2) and the critical appraisal (Section 6.3) make clear, the strongest quantitative claims currently rest on a small number of externally validated comparisons, and several headline results remain preprints awaiting peer review. Neural-ODE models generalize across untested dosing regimens (Lu et al., 2021a); SciML architectures stay interpretable while learning unknown mechanisms (Valderrama et al., 2024); D-PINNs recover population parameter distributions from aggregated data (Tsiros et al., 2026); PINN-based frameworks expose identifiability limitations that classical estimators ignore (Bisht and Agarwal, 2026). For data-scarce, safety-critical applications, and for the mechanism-consistent AI that regulators increasingly expect, these properties are not optional. The trajectory is clear and the pieces are in place; validation will decide the pace.

One further distinction deserves to be explicit. The case for deeper mechanistic coupling made in this review is strongest at the level of mechanism and methodology: mechanism constraints demonstrably improve extrapolation to untested regimens and populations (Lu et al., 2021a; Valderrama et al., 2024), parameter identifiability (Tsiros et al., 2026; Bisht and Agarwal, 2026), physical consistency, and interpretability, and they provide the transparency that regulatory acceptance is beginning to require (US Food and Drug Administration, 2025; European Medicines Agency, 2024). What the current evidence does not support is a claim of clinical superiority in real dosing decisions. No included study prospectively demonstrates that mechanism-constrained models improve dose recommendations, therapeutic-range attainment, safety, or patient outcomes relative to current practice; the tight-coupling advantage remains theoretical and methodological rather than clinical (Figure 9). We regard converting this credible mechanistic argument into demonstrated clinical benefit as the central validation agenda of the field.

Reproduction of the methods reviewed here is already feasible at near-zero cost. The open ecosystem covers every stage of the pipeline, from EHR data (MIMIC-IV), through mechanistic platforms (nlmixr2, OSP/PK-Sim-MoBi), to method code (pharmacoNODE, vancomycin_dose_predictions) and drug-discovery resources (AMPL, TDC, MMPK) (Johnson et al., 2023; ATOM Modeling PipeLine, 2026; Therapeutics Data Commons, 2026; MMPK, 2026; SCAI-Lab MIMIC-IV Data Pipeline, 2026; Lippert et al., 2019; Giacometti et al., 2025; Giacometti, 2026; O'Callaghan, 2026). The challenges ahead are well understood, including data scarcity and heterogeneity, identifiability and uncertainty quantification, multi-drug generalization and special populations, and validation and reporting standards that earn clinical and regulatory trust. Our argument throughout has been that foundation-model scale and mechanism-constrained learning, as exemplified by Foundation PBPK Transformers and physiologically constrained diffusion models (Liu et al., 2026), point to a credible path toward model-informed precision dosing at scale, a promise that has been made for years and may now be within reach. The closing thought is simple. Trust is earned by transparency, black boxes do not dose, and evidence beats assertion.

The next phase of the field will be determined less by algorithmic novelty than by validation discipline and open science, including prospective multi-center studies, standardized reporting (TRIPOD-AI/DECIDE-AI analogues), versioned code and data, and rigorous external validation. Pediatric AED TDM, where data are scarcest and the stakes are highest, is among the most promising proving grounds for these hybrid approaches. The new-generation AEDs (lacosamide, lamotrigine, oxcarbazepine) combine sparse, developmentally heterogeneous sampling with an immediate clinical need, and a multi-drug, mechanism-constrained, closed-loop decision system built on the loose-to-tight coupling framework is a concrete target for the coming years (Chen J. et al., 2025; He et al., 2012; Peng et al., 2014; Winkler et al., 2019; Rodrigues et al., 2017; Chen H. M. et al., 2025). We hope this review provides both a map of the field as it stands and a framework, the loose-to-tight coupling taxonomy, that helps the community navigate the choices ahead, together with the evidence map (Table 2), the pediatric AED gap analysis (Table 4), and the critical appraisal (Section 6.3) that make the field’s claims auditable. Above all, we hope it lands where the clinical reality lives. Kids metabolize differently, samples are scarce, and mechanism is what fills the gap.

It is important to be precise about what the pediatric AED evidence directly supports, as opposed to what the reviewed methods may be able to contribute in the future. Direct pediatric-AED evidence is limited to population PK models (lamotrigine (He et al., 2012); oxcarbazepine-MHD (Peng et al., 2014)) and single-center PPK + ML fusion studies in Chinese pediatric cohorts (Chen et al., 2026; Liu R. et al., 2025; Zhang et al., 2025); none of these integrates mechanism-constrained neural architectures, and none has been externally validated beyond its center of origin (Table 4). The mechanistic-coupling methods reviewed here, concentration forecasting from adult vancomycin and olanzapine cohorts (Khusial et al., 2023; Lu et al., 2021a), PINN-based parameter recovery (Valderrama et al., 2024; Tsiros et al., 2026; Bisht and Agarwal, 2026), and neural-ODE generalization (Lu et al., 2021a), are transferable to pediatric AEDs in principle, but that transfer has not yet been demonstrated on pediatric AED data. We therefore do not claim that a mature, mechanism-constrained AI precision-dosing framework for pediatric AEDs already exists; on the contrary, Table 4 documents that this remains an unmet need. The pediatric AED scenario is a design target and a proving ground, not an existing implementation.
