# Supplementary material for: From time-series deep learning to physics-constrained population pharmacokinetics: AI-driven prediction of drug concentrations and exposure for model-informed dosing
Source: Front Pharmacol. 2026 Sep 22;17:1963219. doi: 10.3389/fphar.2026.1963219 (PMC13639429; doi:10.3389/fphar.2026.1963219)
Supplement: Supplementary file 1 [file Table1.docx]

**Supplementary Appendix S1**

**Literature Search Protocol and Study Eligibility Log**

*Manuscript: “From Time-Series Deep Learning to Physics-Constrained Population Pharmacokinetics: AI-Driven Prediction of Drug Concentrations and Exposure for Model-Informed Dosing”*

This appendix accompanies Section 2 (“Review Methodology”), Figure 2 (search footprint), and Table 1 (evidence map) of the main text. It records the full search protocol, the verbatim search strings, and the study-level eligibility log so that the literature base of the review is fully auditable. All searches were executed on 26 August 2026.

**S1.1 Search strategy overview**

| **Database / platform** | **Coverage** | **Search window** | **Language restriction** | **Records used** |
| --- | --- | --- | --- | --- |
| PubMed (MEDLINE) | Primary index; two exact queries (S1.2) | 1 Jan 2019 – 26 Aug 2026 | English | Stream A n = 1,059; Stream B n = 23 (identification counts, Figure 2) |
| Web of Science Core Collection | Multi-disciplinary; queries adapted (TS=) | 1 Jan 2019 – 26 Aug 2026 | English | Not enumerated individually (purposive selection) |
| Scopus | Multi-disciplinary; queries adapted (TITLE-ABS-KEY) | 1 Jan 2019 – 26 Aug 2026 | English | Not enumerated individually |
| arXiv | Preprint server (CS / q-bio) | 1 Jan 2019 – 26 Aug 2026 | English | Not enumerated individually |
| bioRxiv / medRxiv | Preprint servers (biology / medicine) | 1 Jan 2019 – 26 Aug 2026 | English | Not enumerated individually |
| CNKI | Chinese-language literature; queries adapted with Chinese equivalents | 1 Jan 2019 – 26 Aug 2026 | Chinese | Not enumerated individually |
| Reference-list screening (snowballing) | Backward citation search from all included records | Any date (pre-2019 anchors allowed) | English / Chinese | Foundational anchors [16, 35, 36] and software/data resources (Section 7) |

*Because this is a narrative review with purposive selection (Section 2, main text), identification counts are reported exactly for PubMed (the primary index of both research streams) and qualitatively for the other databases; the selection principles (representativeness, methodological significance, evidence diversity, extractability) are stated in Section 2 of the main text.*

**S1.2 Complete search strings**

**Stream A (time-series / machine learning for drug-concentration prediction), PubMed (executed 26 August 2026):**

("deep learning"[tiab] OR "machine learning"[tiab] OR "neural network"[tiab] OR "long short-term memory"[tiab] OR "neural ordinary differential equation"[tiab] OR "attention"[tiab]) AND (pharmacokinetic[tiab] OR "drug concentration"[tiab] OR "therapeutic drug monitoring"[tiab]) AND (predict[tiab] OR forecasting[tiab] OR dosing[tiab])

**Stream B (physics-constrained modeling), PubMed (executed 26 August 2026):**

("physics-informed"[tiab] OR "scientific machine learning"[tiab] OR "SciML"[tiab] OR "PINN"[tiab] OR "physiologically informed"[tiab] OR "mechanism-constrained"[tiab] OR "neural-ODE"[tiab]) AND (pharmacokinetic[tiab] OR pharmacometric[tiab] OR "drug concentration"[tiab] OR "physiologically based"[tiab] OR "precision dosing"[tiab])

Adaptation to other databases. The same two query families were translated into each database’s syntax: Web of Science (field tag TS=), Scopus (TITLE-ABS-KEY), arXiv/bioRxiv/medRxiv (full-text search), and CNKI (subject terms with Chinese equivalents, e.g., 深度学习, 药代动力学, 治疗药物监测, 精准给药).

Snowballing and hand search. The reference lists of all included records were screened for foundational methodological anchors published before the search window: Bayesian forecasting of individual pharmacokinetics (Sheiner & Beal 1979; 1982) [35, 36], neural ordinary differential equations (Chen et al. 2018) [16], and the origin of the physics-informed neural network framework (Raissi et al. 2019) [9]. Hand search additionally identified the commercial and open MIPD software landscape and the software/data resources inventoried in Section 7.

**S1.3 Eligibility criteria**

Inclusion. Studies were included if they (i) developed or evaluated a model for predicting drug concentrations, pharmacokinetic parameters, or exposure metrics in humans; (ii) used deep learning, machine learning, scientific machine learning, physics-informed, Gaussian process, or hybrid PopPK-ML approaches, alone or combined with population methods; (iii) concerned drugs relevant to therapeutic drug monitoring or precision dosing; and (iv) were published in peer-reviewed journals or posted as preprints, in English or Chinese, within the search window (foundational methods papers outside the window allowed via snowballing).

Exclusion. Conference abstracts, editorials, and letters without quantitative model development; non-human studies; studies not reporting extractable performance metrics or validation design; and duplicate or overlapping reports were excluded. Preprints were not excluded a priori but are flagged as such throughout the evidence map (Table 1) and the critical appraisal (Section 6.3).

**S1.4 Study eligibility log: included primary / applied studies**

| **Study (ref.)** | **Year** | **Peer-review status** | **Drug(s)** | **Evidence level (Figure 9)** |
| --- | --- | --- | --- | --- |
| Khusial et al. [1] | 2023 | Journal | Olanzapine | L1 |
| Lu et al. [6] | 2021 | Journal | Trastuzumab emtansine (T-DM1) | L1 |
| Lu et al. [8] | 2021 | Journal | T-DM1 (concentration–platelet PD) | L1 |
| Ghanbari et al. (Nephrocast-V) [7] | 2025 | Journal | Vancomycin | L3 |
| Chen J et al. [2] | 2025 | Journal | Valproic acid | L1 |
| Chen Z et al. [3] | 2026 | Journal | Tacrolimus | L1 |
| Zhang et al. [5] | 2025 | Journal | Vancomycin AUC24h | L2 |
| Liu R et al. [4] | 2025 | Journal | Voriconazole | L1 |
| Peng et al. [27] | 2014 | Journal | Oxcarbazepine (active metabolite MHD) | L2 |
| Giacometti et al. [23] | 2025 | Journal | Dalbavancin | L2 |
| Raissi et al. [9] | 2019 | Journal | Method foundation (ODE/PDE) | L1 |
| Valderrama et al. [10] | 2024 | Journal | Simulated PK (one-compartment + NN absorption) | L2 |
| Tsiros et al. [11] | 2026 | Journal | Minimal PBPK, monoclonal antibody | L2 |
| Bisht & Agarwal [12] | 2026 | Preprint (arXiv) | Chemotherapy PK (two-compartment + Michaelis-Menten) | L2 |
| Sim2Real-PK [15] | 2026 | Preprint (Research Square) | Ibrutinib; daratumumab | L2 |
| Liu S et al. [13] | 2025 | Preprint (arXiv) | Multi-organ PK | L2 |
| Liu S et al. [14] | 2026 | Preprint (arXiv) | PBPK foundation models | L2 |

*Evidence levels follow the hierarchy of Figure 9: L1 concentration prediction; L2 PK/exposure-parameter estimation; L3 dose recommendation; L4 clinical decision support; L5 prospective clinical-outcome improvement. Classifications were assigned by the two reviewing authors (Y.X., Z.R.) and resolved with B.J. Peer-review status and validation type for each study are recorded in full in Table 1 of the main text.*

**S1.5 Foundational methodological and regulatory anchors**

| **Anchor (ref.)** | **Role in the review** |
| --- | --- |
| Chen et al. (2018) [16] | Origin of neural ordinary differential equations; methodological foundation for continuous-time models (Section 3.2) |
| Karniadakis et al. (2021) [34] | Comprehensive PINN/SciML methodology review; context for Section 4.1 |
| Sheiner & Beal (1979) [35] | Historical Bayesian forecasting foundation for individual pharmacokinetics (Section 3.1) |
| Sheiner & Beal (1982) [36] | MAPB estimation formalism underlying PopPK-based dose adjustment (Section 1) |
| US FDA (2025) [37] | Draft guidance on AI in drug development; risk-based credibility framework (Section 8.4) |
| EMA (2024) [38] | Reflection paper on AI in the medicinal product lifecycle (Section 8.4) |

**S1.6 Notes on identification counts**

(i) PubMed identification counts (Stream A n = 1,059; Stream B n = 23) were obtained from the PubMed E-utilities API on 26 August 2026 and are reproduced in Figure 2. (ii) Counts for the other databases are not enumerated individually because, as a narrative review with purposive selection, we do not report screening-loss arithmetic across every index; the selection principles and the resulting study-level base are fully documented in Section 2, Figure 2, and Table 1. (iii) The bibliometric time series of Figure 8 was retrieved with the same Stream A and Stream B queries per publication year (2019–2026; 2026 partial).
