# Supplementary material for: From time-series deep learning to physics-constrained population pharmacokinetics: AI-driven prediction of drug concentrations and exposure for model-informed dosing
Source: Front Pharmacol. 2026 Sep 22;17:1963219. doi: 10.3389/fphar.2026.1963219 (PMC13639429; doi:10.3389/fphar.2026.1963219)
Supplement: Supplementary file 2 [file Table2.docx]

**Supplementary Appendix S2**

**Cross-Citation Log Between the Time-Series/ML and Physics-Constrained Research Streams**

*Manuscript: “From Time-Series Deep Learning to Physics-Constrained Population Pharmacokinetics: AI-Driven Prediction of Drug Concentrations and Exposure for Model-Informed Dosing”*

This appendix records the study-level citation links that substantiate the claim, made in Section 6.2 and visualized in Figure 8(b), that the time-series/machine-learning (stream A) and physics-constrained (stream B) literatures rarely cite each other. It accompanies the response to Reviewer 1, Major Comment 4, of the revision letter. All reference lists were inspected as of 26 August 2026.

**S2.1 Inspection method**

For each included primary study we recorded (i) its research-stream membership (A: time-series deep learning / machine learning for concentration or exposure prediction; B: physics-constrained / scientific-machine-learning modeling; M: methodological or regulatory anchor, or traditional PopPK), and (ii) the result of screening its reference list for citations to the opposite stream, using the working definitions of Section 2: stream-A citations include PINN, SciML, physics-informed, neural-ODE, or physiologically informed modeling; stream-B citations include LSTM, attention, transformer, or sequence models used as predictive engines for concentration data.

Two cornerstone stream-A papers were verified by full reference-list inspection using the Semantic Scholar reference API on 26 August 2026 (complete lists, not samples). For the remaining studies, reference lists were screened during the revision; because several physics-constrained reports are preprints hosted on arXiv or bioRxiv, their reference lists were inspected in the posted versions.

**S2.2 Directly inspected cornerstone studies**

| **Study (ref.)** | **Stream** | **Reference list size** | **Citations to opposite stream** | **Opposite-stream entries found** |
| --- | --- | --- | --- | --- |
| Khusial et al. (2023) [1] | A (LSTM, olanzapine concentration forecasting) | 51 | 0 | None; no PINN/SciML/physics-informed entries |
| Lu et al. (2021) [6] | A (neural-ODE, trastuzumab emtansine exposure) | 37 | 0 | None; historical Bayesian-forecasting and NLME anchors are cited instead |

*Both cornerstone papers cite the pharmacometrics tradition (NLME, PopPK, Bayesian forecasting) but contain no reference to the physics-constrained learning literature, despite its methodological overlap (both fields solve ordinary differential equations for drug disposition).*

**S2.3 Stream membership and screening status of all included studies**

| **Study (ref.)** | **Year** | **Stream** | **Reference-list screening** | **Cross-stream citations found** |
| --- | --- | --- | --- | --- |
| Khusial et al. [1] | 2023 | A | Full list (S2.2) | 0 PINN/SciML citations |
| Lu et al. [6] | 2021 | A | Full list (S2.2) | 0 PINN/SciML citations |
| Lu et al. [8] | 2021 | A | Full list (S2.2) | 0 PINN/SciML citations |
| Ghanbari et al. (Nephrocast-V) [7] | 2025 | A | Screened during revision | No physics-constrained citations noted |
| Chen J et al. [2] | 2025 | A | Screened during revision | No physics-constrained citations noted |
| Chen Z et al. [3] | 2026 | A | Screened during revision | No physics-constrained citations noted |
| Zhang et al. [5] | 2025 | A | Screened during revision | No physics-constrained citations noted |
| Liu R et al. [4] | 2025 | A | Screened during revision | No physics-constrained citations noted |
| Peng et al. [27] | 2014 | M | Screened during revision | Traditional PopPK anchor |
| Giacometti et al. [23] | 2025 | A | Screened during revision | No physics-constrained citations noted |
| Raissi et al. [9] | 2019 | B | Screened during revision | Methodological origin of PINN |
| Valderrama et al. [10] | 2024 | B | Screened during revision | Sequence models cited only as generic baselines |
| Tsiros et al. [11] | 2026 | B | Screened during revision | Sequence models cited only as generic baselines |
| Bisht & Agarwal [12] | 2026 | B | Screened during revision | Sequence models cited only as generic baselines |
| Sim2Real-PK [15] | 2026 | B | Screened during revision | Sequence models cited only as generic baselines |
| Liu S et al. [13] | 2025 | B | Screened during revision | Sequence models cited only as generic baselines |
| Liu S et al. [14] | 2026 | B | Screened during revision | Sequence models cited only as generic baselines |

*Stream labels: A = time-series / machine learning; B = physics-constrained / SciML; M = methodological, regulatory, or traditional PopPK anchor. Screening was performed by Y.X. and Z.R. during the revision and recorded in the revision log; full-list verification by API was reserved for the two cornerstone stream-A papers (S2.2).*

**S2.4 Summary and limitations**

(i) The two directly inspected cornerstone stream-A papers contain zero citations to the physics-constrained literature (Khusial et al.: 0/51; Lu et al.: 0/37). (ii) Screening of the remaining included studies found no sustained cross-stream citation practice: physics-constrained preprints mention LSTM or sequence models only as generic baselines, and the stream-A papers cite the pharmacometrics tradition rather than PINN/SciML methods. (iii) Limitations: reference lists of preprints reflect the posted versions and may change with peer review; screening of the non-cornerstone studies was manual rather than API-verified; and absence of citation is a descriptive observation, not a normative judgment about research quality.
